# Hyaluronan-based graft copolymers bearing aggregation-induced emission fluorogens†

**DOI:** 10.1039/c7ra12543g

**Published:** 2018-02-06

**Authors:** Andrea Cappelli, Marco Paolino, Annalisa Reale, Vincenzo Razzano, Giorgio Grisci, Germano Giuliani, Alessandro Donati, Claudia Bonechi, Stefania Lamponi, Raniero Mendichi, Salvatore Battiato, Filippo Samperi, Francesco Makovec, Mariano Licciardi, Lorenzo Depau, Chiara Botta

**Affiliations:** Dipartimento di Biotecnologie, Chimica e Farmacia and European Research Centre for Drug Discovery and Development, Università degli Studi di Siena Via A. Moro 2 53100 Siena Italy andrea.cappelli@unisi.it +39 0577 234320; Istituto per lo Studio delle Macromolecole (CNR) Via A. Corti 12 20133 Milano Italy; Istituto per i Polimeri, Compositi e Biomateriali (IPCB) U.O.S. di Catania, CNR Via Gaifami 18 95126 Catania Italy; Rottapharm Biotech Via Valosa di Sopra 9 20900 Monza Italy; Dipartimento di Scienze e Tecnologie Biologiche, Chimiche e Farmaceutiche (STEBICEF), Università degli Studi di Palermo Via Archirafi 32 90123 Palermo Italy; Mediterranean Center for Human Health Advanced Biotechnologies (CHAB), University of Palermo Italy; Dipartimento di Biotecnologie Mediche, Università degli Studi di Siena Via A. Moro 2 53100 Siena Italy

## Abstract

In order to develop a technology platform based on two natural compounds from biorenewable resources, a short series of hyaluronan (HA) copolymers grafted with propargylated ferulic acid (HA–FA–Pg) were designed and synthesized to show different grafting degree values and their optical properties were characterized in comparison with reference compounds containing the same ferulate fluorophore. Interestingly, these studies revealed that the ferulate fluorophore was quite sensitive to the restriction of intramolecular motion and its introduction into the rigid HA backbone, as in HA–FA–Pg graft copolymers, led to higher photoluminescence quantum yield values than those obtained with the isolated fluorophore. Thus, the propargyl groups of HA–FA–Pg derivatives were exploited in the coupling with oleic acid through a biocompatible nona(ethylene glycol) spacer as an example of the possible applications of this technology platform. The resulting HA–FA–NEG–OA materials showed self-assembling capabilities in aqueous environment. Furthermore, HA–FA–NEG–OA derivatives have been shown to interact with phospholipid bilayers both in liposomes and living cells, retaining their fluorogenic properties and showing a high degree of cytocompatibility and for this reason they were proposed as potential biocompatible self-assembled aggregates forming new materials for biomedical applications.

## Abstract 2

A new technology platform has been developed with hyaluronan playing the role of the macromolecular carrier and ferulate the central role of natural small molecule fluorogenic clickable linker.

## Graphical abstract

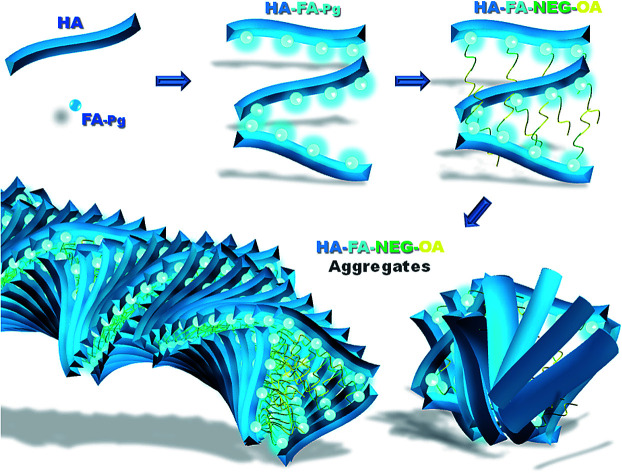

## Introduction

The hydroxycinnamic derivative ferulic acid (FA, 4-hydroxy-3-methoxycinnamic acid) is largely distributed in the plant cellular wall,^1^ where it is linked by means of an ester bond to primary alcohol groups of arabinose side chains of arabinoxylans. The FA residues inserted in the cell wall of polysaccharides play the crucial role of cross-linking polysaccharides and proteins during cell wall synthesis.^1–4^ Furthermore, FA residues can be transformed into a large variety of dehydrodiferulate dimers capable of bridging different polysaccharide chains by peroxidase-mediated oxidative coupling (or dimerization and photoisomerism by UV light).^1–4^FA also possesses interesting fluorescence properties, with absorption/emission features affected by both solvent polarity and pH.^5^FA has been observed to be an inhibitor of beta amyloid pathological aggregation^6^ and has been incorporated into biodegradable polymers as a pendant group to enhance antioxidant properties for tissue engineering applications.^7–9^ Polymers containing FA in the main chain have also been proposed to obtain materials for sensing and/or imaging applications.^10–16^ Aggregation induced emission (AIE) in biocompatible fluorophores have gained much attention in recent years since AIE compounds, being weakly emissive or non-emissive in solution due to free intramolecular motions, become good emitters upon aggregation and therefore represent a class of excellent fluorescent bioprobes.^17^

The glycosaminoglycan hyaluronic acid (HA, hyaluronan) plays important roles in human body. Among them, HA forms a pericellular coat playing a pivotal role in the early stages of cell adhesion^18^ by interacting with CD44 receptor.^19,20^ This receptor is an ubiquitous protein possessing an extracellular portion called hyaluronan binding domain and has been described to be expressed in high density in tumor tissues.^21^

**Fig. 1:**
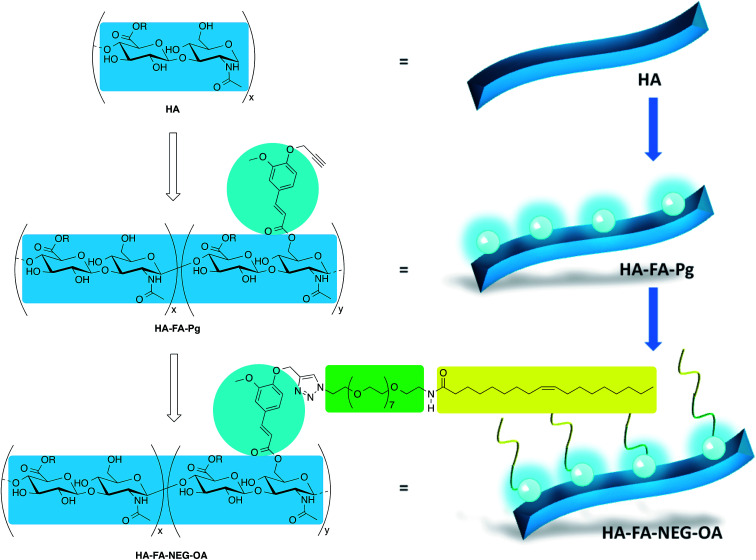
Design of multi-component material HA–FA–NEG–OA based on hyaluronic acid (HA, light blue), ferulic acid (FA, cyan), nona(ethylene glycol) (NEG, green), and oleic acid (OA, yellow). Substituents: R = H or C_2_H_5_.

In our previous studies, the HA backbone was grafted with variable densities of FA residues to afford HA–FA graft copolymers in the aim of combining the intriguing properties of the two natural compounds.^22,23^ Furthermore, a tri-component polymer brush was designed by functionalization of a polybenzofulvene derivative with nona(ethylene glycol) (NEG) side chains terminated with low molecular weight hyaluronic acid macromolecules.^24,25^ This material was prepared by means of a convergent approach employing a copper(i)-catalyzed azide–alkyne 1,3-dipolar cycloaddition (CuAAC) of the suitable polybenzofulvene derivative (the azide component) with the hyaluronan derivative HA–FA–Pg (Fig. 1) bearing propargyl groups bound to HA backbone through FA fluorophores.^24^

In order to develop a technology platform based on two natural compounds from biorenewable resources, we prepared a short series of hyaluronan-based graft copolymers (HA–FA–Pg) showing different grafting degree (GD) values and we studied their optical properties. In the attempt to demonstrate the usefulness of the technology platform, the propargyl groups of HA–FA–Pg derivatives were exploited in the CuAAC coupling with alpha-azido-omega-oleic amide nona(ethylene glycol) (compound 3, azido-NEG–OA) to obtain amphiphilic HA derivatives (*i.e.*HA–FA–NEG–OA). The obtained HA–FA–NEG–OA showed potential self-assembling abilities in aqueous environment and for this reason was herein exhaustively characterized and proposed as biocompatible material with high potentiality in biomedical applications (*i.e.* in drug delivery).

## Experimental section

### Synthesis and characterization

All reagents and solvents were purchased from Sigma-Aldrich and were used as received, with the exceptions noted. Merck TLC aluminum sheets, silica gel 60 F_254_ were used for TLC. NMR spectra were recorded with either a Bruker DRX-400 Avance, a Bruker DRX-500 AVANCE, a Bruker AMX-600 Avance, or a Bruker Avance 900 spectrometer, working at 900.13 MHz frequency and equipped with a cryogenically cooled probe in the indicated solvents (TMS as internal standard).

### General procedure for the preparation of HA–FA–Pg graft copolymers

Into a two-necked round-bottomed 50 mL flask, a mixture of low molecular weight HA (8700 Da, Biophil Italia SpA, see the amounts in Table 1) in formamide (10 mL) was heated from 23 to 50 °C till the complete dissolution was obtained. The resulting solution was then cooled to room temperature and triethylamine (TEA, 0.37 mL, 2.66 mmol) and 1^24^ (see the amounts in Table 1) were added in sequence. After stirring the reaction mixture overnight at room temperature, a 5% NaCl solution (5.0 mL) was added and the resulting mixture was stirred at room temperature for additional 10–15 min. The expected graft copolymer derivative was obtained by treatment of the mixture with acetone (40 mL) and purified by washing four times with the same solvent. The final solid was dried under reduced pressure to afford the expected HA–FA–Pg graft copolymer as a white solid. ^1^H NMR (600 MHz, D_2_O): see Fig. 3; ^13^C NMR (125 MHz, D_2_O): see Fig. 4.

**Table 1:** Reaction parameters in the functionalization of HA polymer to HA–FA–Pg graft copolymers with imidazolide 1

Entry	Copolymer	HA (g)	HA (mmol)	1/HA ratio (%)	Grafting degree^a^ (%)	Convers.^b^ (%)
1	HA–FA–Pg-01	0.50	1.32	25	20	80
2	HA–FA–Pg-02	0.50	1.32	25	18	72
3	HA–FA–Pg-03	0.50	1.32	25	20	80
4	HA–FA–Pg-1F	0.39	1.03	50	33	66
5	HA–FA–Pg-2F	0.50	1.32	50	35	70
6	HA–FA–Pg-3F^c^	1.0	2.64	25	20	80
7	HA–FA–Pg-4F	1.0	2.64	12.5	10	80

a A rough estimate of the grafting degree was made by ^1^H NMR spectroscopy after hydrolysis with NaOD in D_2_O as described in ref. 22.

b The conversion into ferulate was calculated from the substitution degree and stoichiometric ratio 1/HA.

c See ref. 24.

**Fig. 2:**
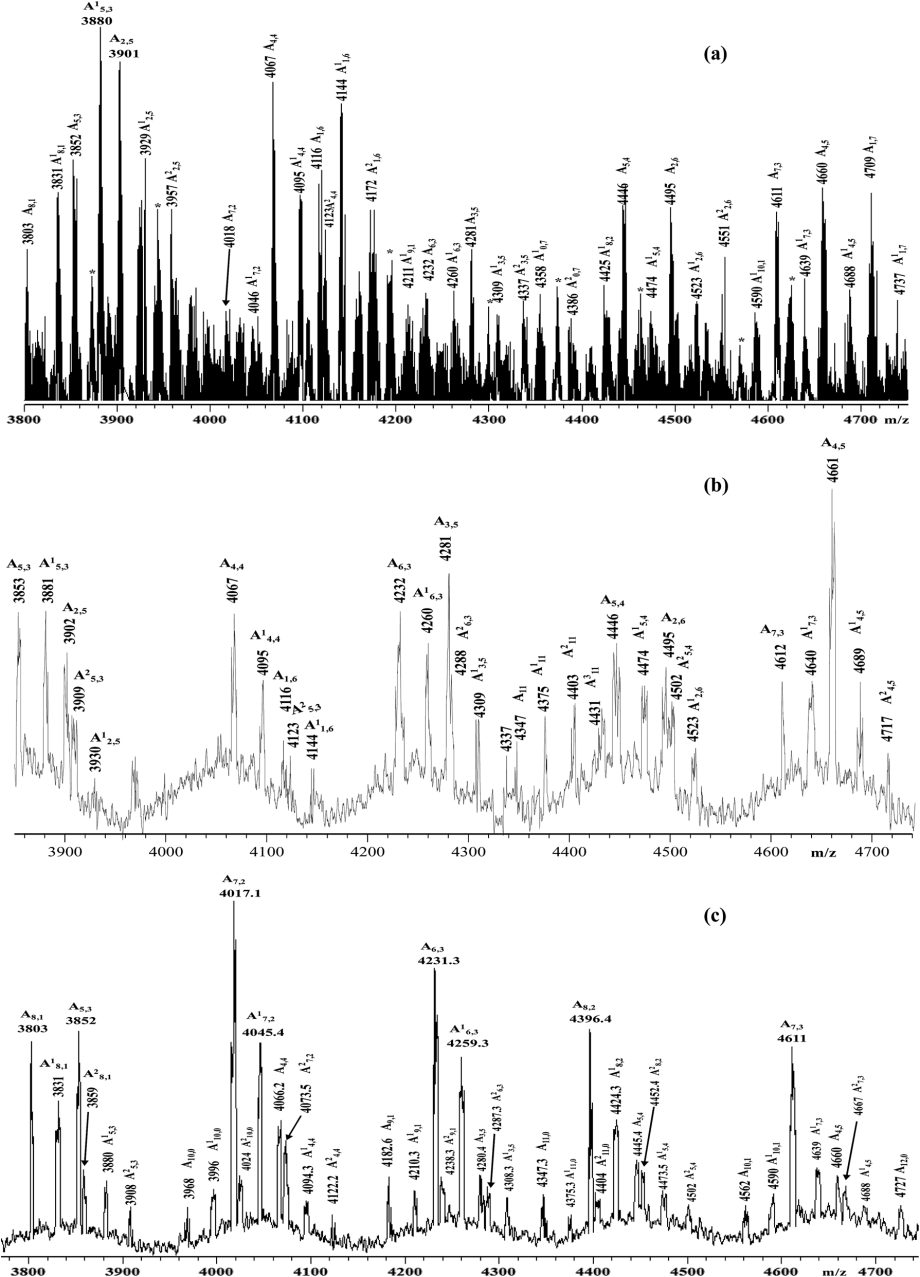
Enlarged sections of the MALDI-TOF mass spectra (negative ions) of the samples: (a) HA–FA–Pg-2F; (b) HA–FA–Pg-3F; (c) HA–FA–Pg-4F.

**Fig. 3:**
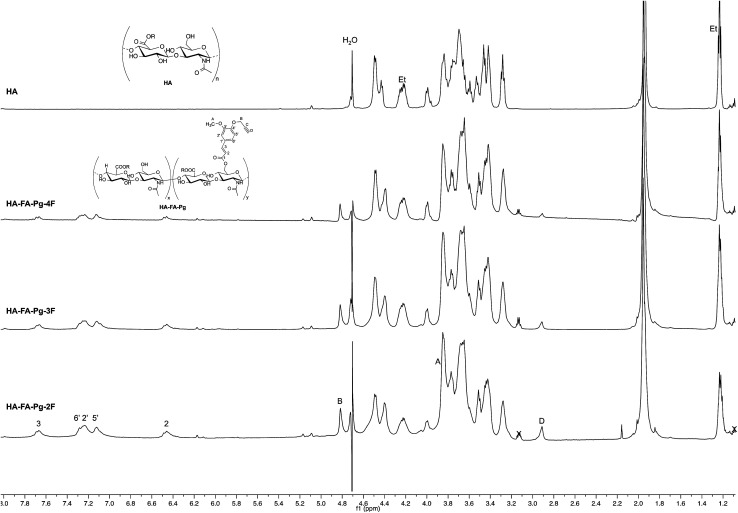
Comparison of ^1^H NMR spectra obtained with some selected HA–FA–Pg derivatives (D_2_O, 600 MHz, with water suppression) with that obtained with starting HA sample. In the spectrum of HA, Et labels indicate the signals of ethyl groups of the monomeric units showing R = C_2_H_5_.

**Fig. 4:**
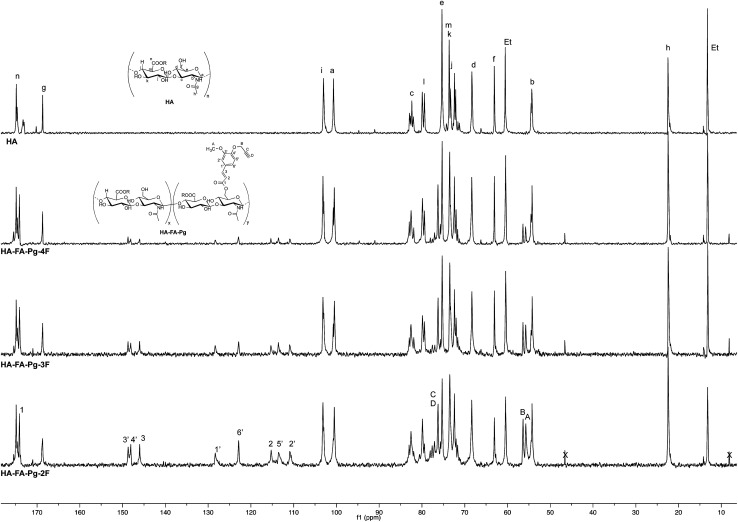
Comparison of ^13^C NMR spectra obtained with some selected HA–FA–Pg derivatives (D_2_O, 125 MHz) with that obtained with starting HA sample. In the spectrum of HA, Et labels indicate the signals of ethyl groups of the monomeric units showing R = C_2_H_5_.

### Synthesis of HA–FA–NEG–OA–4 material

**Fig. 5:**
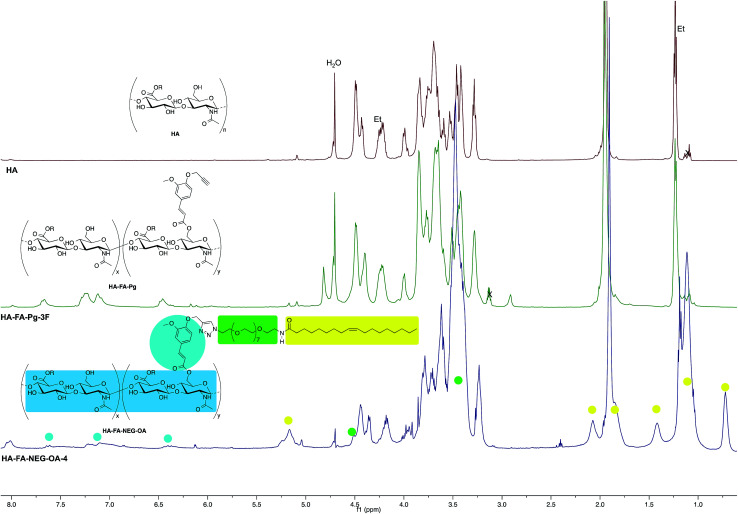
Comparison of ^1^H NMR spectrum obtained with HA–FA–NEG–OA–4 derivative (D_2_O, with water suppression) with those obtained with starting HA–FA–Pg-3F and HA samples. In the spectrum of HA, Et labels indicate the signals of ethyl groups of the monomeric units showing R = C_2_H_5_.

**Fig. 6:**
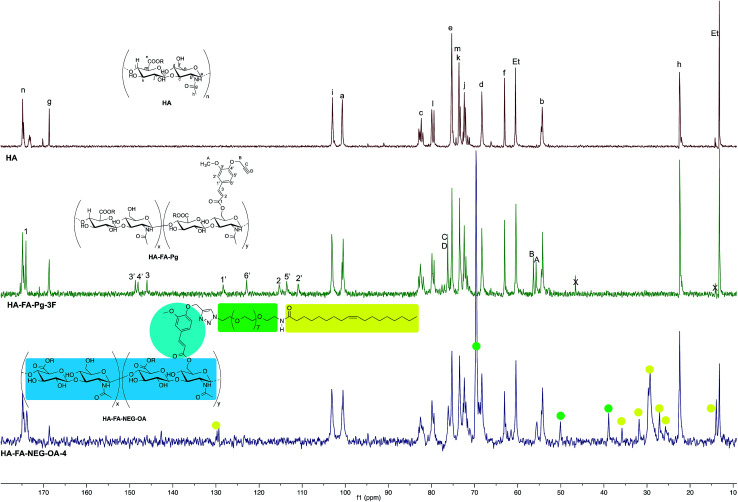
Comparison of ^13^C NMR spectrum obtained with HA–FA–NEG–OA–4 derivative (125 MHz, D_2_O) with those obtained with starting HA–FA–Pg-3F and HA samples. In the spectrum of HA, Et labels indicate the signals of ethyl groups of the monomeric units showing R = C_2_H_5_.

Under an inert atmosphere, a 10 mL flask was charged with *tert*-butanol (2.0 mL), water (2.0 mL), and a solution of CuSO_4_ pentahydrate (12.5 mg, 0.050 mmol) in 0.50 mL of water. A 1 M solution of sodium ascorbate in water (0.50 mL) was then added and 1.0 mL of the resulting mixture was used as the catalyst. A mixture of 3 (37 mg, 0.0526 mmol) and HA–FA–Pg-3F graft copolymer (100 mg) in water (5.0 mL) was treated with the catalyst solution (1.0 mL) and the reaction mixture was stirred at room temperature for 4 h and then treated with QUADRASIL MP (200 mg). After filtration the solution was concentrated under reduced pressure. Purification of the residue by washing with acetone gave HA–FA–NEG–OA–4 material, which was dried under reduced pressure to obtain as a light brown glassy solid (75 mg). ^1^H NMR (500 MHz, D_2_O): Fig. 5; ^13^C NMR (125 MHz, D_2_O): Fig. 6.

### Synthesis of HA–FA–NEG–OA–8 material

HA–FA–NEG–OA–8 material was prepared by applying the above procedure to HA–FA–Pg-2F graft copolymer (100 mg) and 3 (54 mg, 0.0768 mmol) at room temperature for 6 h. HA–FA–NEG–OA–8 was obtained as a brown glassy solid (80 mg). ^1^H NMR (500 MHz, D_2_O): see Fig. ESI-1.†

### SEC-MALS

The molecular characterization was performed by a multi-angle laser light scattering (MALS) detector on line to a size exclusion chromatography (SEC or GPC) system. The SEC-MALS multi-detector system consisted of an Alliance 2695 chromatograph from Waters (USA) with two on-line detectors: a MALS Dawn DSP-F photometer from Wyatt (USA) and a 410 differential refractometer (DRI) from Waters as concentration detector. In order to optimize sample solubility, regular elution, and SEC fractionation, an unusual solvent mixture [*i.e.* 0.2 M aqueous NaCl solvent and dimethyl sulfoxide (DMSO) organic solvent (50 : 50 w/w)] was used. The SEC experimental conditions were the following: two Polargel SEC columns (M and L, 5 μm of particle size) from Polymers Laboratories (UK), 35 °C of temperature, 0.5 mL min^−1^ of flow rate, about 5 mg mL^−1^ of sample concentration.

The MALS calibration constant was calculated using toluene as standard by assuming a Rayleigh factor of 1.406 × 10^−5^ cm^−1^. The MALS angular normalization was performed by measuring the scattering intensity of a concentrated solution of a pullulan polysaccharide with narrow molecular weight distribution (MWD, *M*_p_ = 12 kg mol^−1^, *M*_w_/*M*_n_ < 1.03, *R*_g_ = ≈2.1 nm) assumed to act as an isotropic scatterer. It is known that the on-line MALS detector measures, for each polymeric fraction eluted from the SEC columns, the molecular weight (*M*) and when the angular dependence of the scattered light is experimentally measurable also the molecular size generally known as radius of gyration (*R*_g_). The SEC-MALS system was described in detail elsewhere.^26,27^

The differential refractive index increment of the polymers with respect to the solvent mixture was measured off-line by a Chromatix KMX-16 differential refractometer.

### MALDI-TOF MS

MALDI TOF MS (matrix assisted laser desorption/ionization time of flight mass spectrometry) mass spectra were carried out using a 4800 Proteomic Analyzer (Applied Biosystems) MALDI-TOF/TOF instrument equipped with a Nd:YAG laser at a wavelength of 355 nm with <500 ps pulse and 200 Hz firing rate, the acceleration voltage was set at 20 kV. The irradiance was maintained slightly above the threshold, to obtain a mass resolution of about 1000–2000 fwhm; isotopic resolution was observed throughout the entire mass range detected (from *m*/*z* 1000 up to *m*/*z* 6000). External calibration was performed using an Applied Biosystems calibration mixture consisting of polypeptides with different molecular weight values. Mass accuracy was about 150–200 ppm. All measurements were performed in negative ion mode; approximately 1500 laser shots were accumulated for each mass spectrum. The best spectra were recorded using 2,5-dihydroxybenzoic acid (2,5-DHB) or *trans*-2-[3-(4-*t*-butyl-phenyl)-2-methyl-2-propenylidene]malononitrile (DCTB) as matrices (0.1 M in CH_3_CN/CH_3_OH 1/1 v/v). Polymer samples were solubilized in the CH_3_CN/CH_3_OH (1 : 1 v/v) or CHCl_3_/CH_3_OH (2 : 1 v/v) solvent mixtures with a concentration of about 2 mg mL^−1^. Samples for MALDI analysis were prepared by the dried-droplet method, in which a mixture of matrix and sample (0.3 μL) was deposited onto the target plate and dried at room temperature under inert atmosphere (N_2_ flow).

### Dynamic light scattering (DLS) analysis and *ζ* potential measurements

The mean diameter, width of distribution (polydispersity index, PDI), and *ζ* potential of the nanoparticles were measured at 25 °C using a Zetasizer NanoZS instrument fitted with a 532 nm laser at fixed scattering angle of 173°. The intensity-average hydrodynamic diameter (size in nm) and PDI of the materials were measured in double distilled water. The *ζ* potential (mV) was calculated from the electrophoretic mobility using the Smoluchowski relationship and assuming that *K* × *a* ≫ 1 (where *K* and *a* are the Debye–Hückel parameter and particle radius, respectively). Each experiment was performed in triplicate.

### Synthesis of liposomes

Empty 1,2-dioleoyl-*sn*-glycerophosphocholine (DOPC)/1,2-dioleoyl-*sn*-glycerophosphoethanolamine (DOPE) liposomes were prepared at 1 : 1 mol ratio with a total lipid concentration of 1.0 × 10^−2^ M. HA–FA–NEG–OA–8 material was loaded in zwitterionic liposomes. Liposomes were prepared in a round bottom vial by mixing the appropriate amounts of stock solutions, which were 3 × 10^−2^ M in chloroform for lipids, and 5 mg mL^−1^ in water for HA–FA–NEG–OA–8. A dry lipid film was obtained by evaporating the solvent under vacuum overnight. Rehydrating with Milli-Q grade H_2_O yielded multilamellar dispersion. Upon vortexing, multilamellar vesicles were obtained, which were then submitted to nine freeze/thaw cycles to improve the homogeneity of the size distribution in the final suspension. Liposomes were subsequently reduced in size and converted to unilamellar vesicles by extrusion through 100 nm polycarbonate membranes. Twenty-seven extrusions were performed with the LiposoFast apparatus (Avestin, Ottawa, Canada). All liposomes were stored at 4 °C.

### Optical spectroscopy

The UV-vis measurements were performed with a Perkin Elmer Lambda 900 spectrometer. PL spectra were obtained with a Nanolog spectrofluorimeter equipped with a Synapse QExtra CCD. The spectra were corrected for the instrument response. Photoluminescence (PL) quantum yield (QY) values of solutions were obtained by using quinine sulfate as the reference. PLQY of solid powders were measured with a SPEX 270 M monochromator with a home-made integrating sphere according to the procedure reported elsewhere.^28^

### *In vitro* cell studies

In order to evaluate the *in vitro* cytotoxicity of HA–FA–NEG–OA materials, the direct contact tests, proposed by ISO 10995-5, Biological evaluation of medical devices – Part 5: Tests for cytotoxicity: *in vitro* methods was used. This test is suitable for samples with various shapes, sizes or physical status (*i.e.* liquids or solids). The evaluation of *in vitro* acute toxicity does not depend on the final use for which the product is intended, and the document ISO 10995-5:2009 recommends many cell lines from American Type Collection. Among them, to test HA–FA–NEG–OA cytotoxicity, NIH3T3 mouse fibroblasts were chosen (see ESI† for the details).^29^ The direct contact test was also used for cytocompatibility evaluation by using Human Chondrocyte (HC). HC were propagated in chondrocyte growth medium and NIH3T3 in DMEM supplemented with 10% fetal calf serum, 1% l-glutamine–penicillin–streptomycin solution, and 1% MEM non-essential amino acid solution, and incubated at 37 °C in a humidified atmosphere containing 5% CO_2_. Once at confluence, the cells were washed with 0.1 M PBS, separated with trypsin–EDTA solution and centrifuged at 1000 rpm for 5 minutes. The pellet was re-suspended in complete medium (dilution 1 : 15).

PANC-1 (human pancreas adenocarcinoma) cells for confocal microscopy studies were obtained from American Type Collection and cultured as described above.

### Fluorescence imaging

The internalization of HA–FA–NEG–OA materials in NIH3T3 cells was analyzed by fluorescence microscopy, and in PANC-1 cells by confocal microscopy.

#### Fluorescence microscopy

Cover slips were deposited on the wells of a 6-well plate. 1.5 × 10^5^ cells were seeded in each well. Cells were incubated with 7 × 10^−2^ mg mL^−1^ concentration of HA–FA–NEG–OA–4 for 15 min at 37 °C. Then, cells were washed with PBS, fixed with 4% paraformaldehyde for 10 min at room temperature and visualized using an inverted fluorescent microscope (Axiovert 200, Zeiss, Germany).

#### Confocal microscopy

Cover glass slides were deposited on the bottom of the wells of a 24-well plate. 6 × 10^4^ PANC-1 cells were seeded in each well. After 24 h, cells were incubated with 3 × 10^−2^ mg mL^−1^ of HA–FA–NEG–OA materials for 30 min at 37 °C. The cells were then incubated with medium for 1, 2 or 4 h at 37 °C. Then cells were fixed with 4% paraformaldehyde for 10 min at room temperature and stained with Lectin-FITC (1 : 3000 in PBS–BSA 1%) for 10 min at room temperature in the dark. After each step were performed two washes with PBS. Glasses were then mounted with Mowiol and visualized using a confocal microscope (SP5, Leica, Germany).

## Results and discussion

### Synthesis of HA–FA–Pg and HA–FA–NEG–OA derivatives

**Scheme 1:**
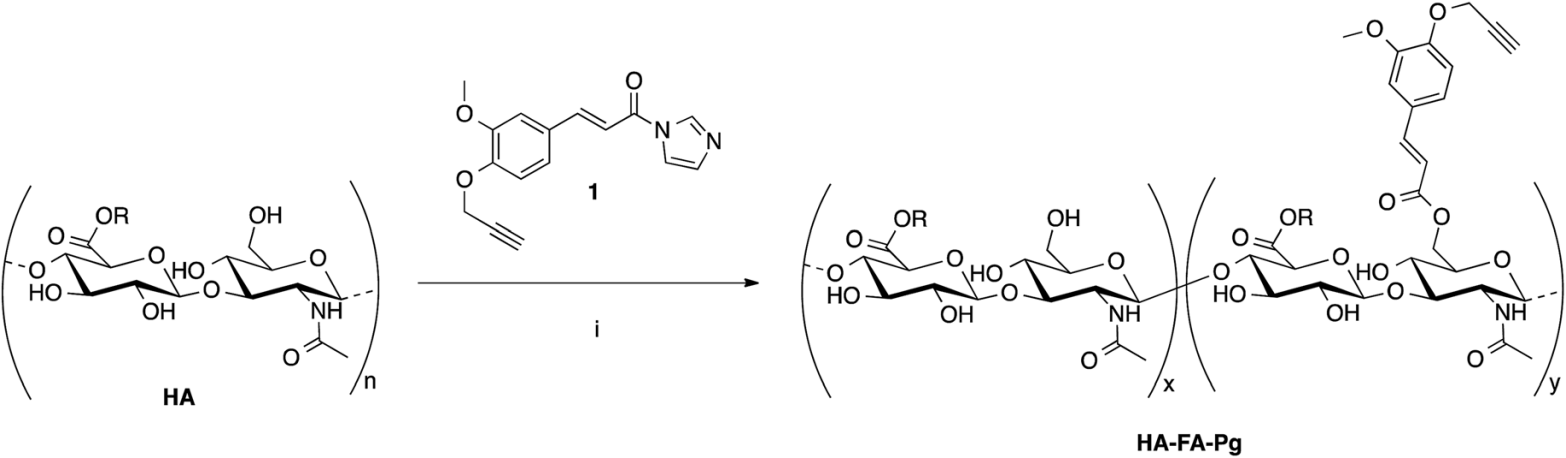
Preparation of the HA–FA–Pg derivatives. Reagents: (i) HCONH_2_, TEA. Substituents: R = H or C_2_H_5_.

The hyaluronan derivatives HA–FA–Pg were prepared by reaction of low molecular weight HA with imidazolide 1^24^ as reported in Scheme 1.

The functionalization reaction was carried out in formamide as the solvent and in the presence of triethylamine (TEA) as the base to afford the desired graft copolymer HA–FA–Pg.^24^ The stoichiometric ratio between 1 and HA (*i.e.*1/HA ratio, Table 1) was varied from 50 to 12.5% with a number of replicates in 1/HA ratios of 25% and 50% in the aim of evaluating the reproducibility of the molar grafting degree (GD) observed. The isolation of the copolymer from the reaction mixture was performed by precipitation with acetone to obtain samples as white solids.

The analysis of the data reported in Table 1 demonstrate that GD value can be regulated in the range 10–35% by using the correct 1/HA stoichiometric ratio, with high and quite reproducible conversion values (*i.e.* 60–80%).

**Scheme 2:**
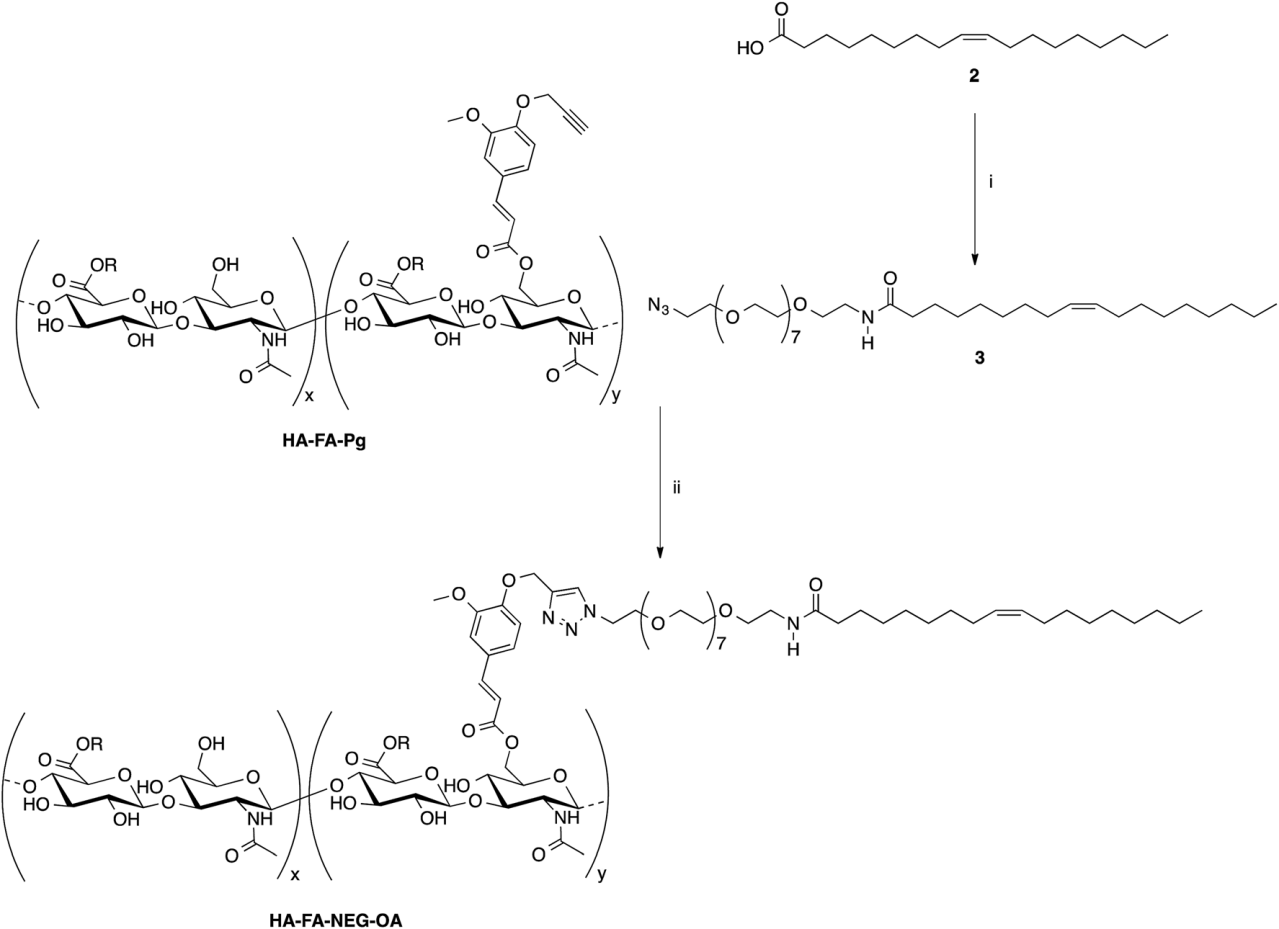
Click chemistry functionalization of HA–FA–Pg with azido-NEG–OA leading to HA–FA–NEG–OA. Reagents: (i) CDI, THF, N_3_CH_2_CH_2_(OCH_2_CH_2_)_8_NH_2_; (ii) CuSO_4_, sodium ascorbate, *tert*-BuOH, H_2_O. Substituents: R = H or C_2_H_5_.

Some selected HA–FA–Pg samples were then used in the CuAAC coupling with alpha-azido-omega-oleic amide nona(ethylene glycol) (azido-NEG–OA, 3) to obtain HA derivatives (*i.e.*HA–FA–NEG–OA) as depicted in Scheme 2.

The copper(i) catalyst was generated *in situ* with CuSO_4_/sodium ascorbate in order to perform the CuAAC coupling under very mild conditions. The coupling reaction was carried out by using two HA–FA–Pg samples showing different grafting degrees (*i.e.* about 20% corresponding to four propargyl groups per HA macromolecule in HA–FA–Pg-3F, and about 35% corresponding to eight propargyl groups per HA macromolecule in HA–FA–Pg-2F) in the aim of obtaining HA–FA–NEG–OA derivatives bearing different densities of OA side chains pending from the HA backbone (*i.e.* about four in HA–FA–NEG–OA–4, and about eight in HA–FA–NEG–OA–8).

**Scheme 3:**
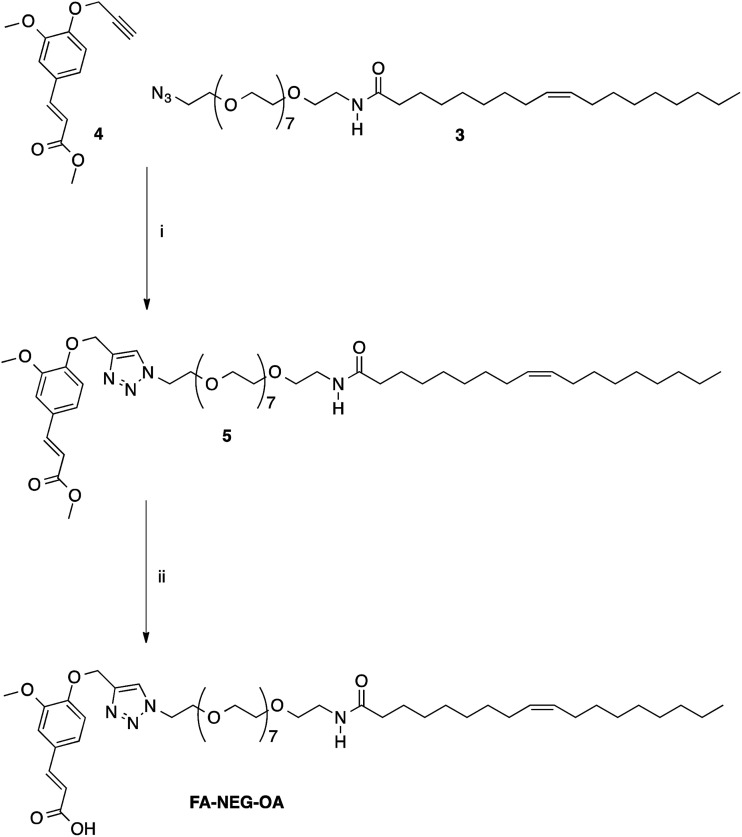
Synthesis of model compounds 5 and FA–NEG–OA. Reagents: (i) CuSO_4_, sodium ascorbate, *tert*-BuOH, H_2_O; (ii) NaOH, H_2_O, C_2_H_5_OH.

Finally, compound FA–NEG–OA and its methyl ester precursor 5 were synthesized as described in Scheme 3 in order to be used as model compounds.

### Molecular characterization of HA–FA–Pg derivatives

The molecular characterization of HA–FA–Pg graft copolymers and of the starting HA was performed by means of a multi-angle laser light scattering (MALS) absolute detector on-line to a size exclusion chromatography (SEC) system by using a suitable solvent mixture [*i.e.* 0.2 M NaCl–DMSO (80 : 20)] as the mobile phase. The most important SEC-MALS results obtained [*i.e.* the molecular weight of the peak of the chromatogram (*M*_p_), the weight-average of the molecular weight (*M*_w_), and the polydispersity index *M*_w_/*M*_n_ where *M*_n_ denotes the numeric-average of the molecular weight] are summarized in Table 2. The table also reports the recovered mass that is the fraction of polymeric sample eluting from the SEC columns, the d*n*/d*c*, and the grafting degree values.

**Table 2:** Macromolecular features of starting HA and HA–FA–Pg graft copolymers

Sample	d*n*/d*c* (mL g^−1^)	*M*_p_ (kg mol^−1^)	*M*_w_ (kg mol^−1^)	*M*_w_/*M*_n_	Rec. mas.^a^ (%)	Grafting^b^ (%)
HA	0.116	8.5	8.7	1.53	70	0
HA–FA–Pg-1F	0.125	8.4	7.3	1.40	61	33
HA–FA–Pg-2F	0.125	7.0	6.3	1.34	59	35
HA–FA–Pg-3F	0.122	6.8	6.5	1.45	56	20
HA–FA–Pg-4F	0.120	7.0	6.7	1.22	66	10

a Recovered mass: the fraction of the polymeric sample eluting from the SEC columns.

b A rough estimate of the grafting degree was made by ^1^H NMR spectroscopy after hydrolysis with NaOD in D_2_O as described in ref. 22.

In consideration of the complex copolymer structure and the unusual SEC solvent mixture, the recovered mass values of the samples were relative high and substantially adequate for estimating the whole MWD of the copolymers, particularly after the derivatization reactions. Consequently, the SEC-MALS results in Table 2 provide interesting and very useful information for the native HA and the HA–FA–Pg derivatives.

The molecular weight of the starting HA sample was relatively low for a HA polysaccharide (*M*_w_ average about 8.7 kg mol^−1^) and the MWD was substantially broad (*M*_w_/*M*_n_ about 1.5). On the whole, in consequence of grafting the MWD of HA–FA–Pg derivatives change only a little (*i.e.* the *M*_w_ average decrease from 8.7 kg mol^−1^ of the starting HA to 6.3 kg mol^−1^ of the HA–FA–Pg-2F derivative showing 35% of grafting). During derivatization reactions two molecular weight tendencies are simultaneously working in opposite ways: (1) a molecular weight decrease produced by an eventual degradation; (2) a molecular weight increase produced by the grafting. The data reported in Table 2 demonstrated that degradation of HA starting biopolymer is relatively low also for high level of grafting. Furthermore, the d*n*/d*c* values appeared to be quite well related with the grafting degree and this result substantially confirmed the estimation of the grafting.

Functionalized HA–FA–Pg samples (*i.e.*HA–FA–Pg-2F, HA–FA–Pg-3F, and HA–FA–Pg-4F) were characterized by MALDI-TOF MS in negative-ion mode using DHB or DCTB as the matrix. The best mass resolved mass spectra of the HA–FA–Pg-3F and HA–FA–Pg-4F samples were recorded using the DHB as the matrix, and the DCTB one for the HA–FA–Pg-2F derivative. As previously reported,^24^ the mass spectrum of starting HA showed, in the mass range *m*/*z* 2500–10 000 Da, a series of clusters corresponding to the even-numbered HA oligosaccharides (7- to 23-mers, species A_*n*_). On the other hand, complex mass spectra were obtained in the case of the hyaluronan graft copolymers HA–FA–Pg-2F, HA–FA–Pg-3F, and HA–FA–Pg-4F, which shows a series of cluster of peaks in the mass range *m*/*z* 3500–10 000. Enlarged sections of these mass spectra are reported in Fig. 2 together with the structural assignment. The analysis of the spectra suggested that the most intense peaks belong to the expected graft copolymer chains (species A_*x*,*y*_). In particular, most of the copolymer chains present along the backbone: 6–8 ferulate residues in the case of HA–FA–Pg-2F, 4–5 ferulate residues in the case of HA–FA–Pg-3F, 2–3 ferulate residues in the case of HA–FA–Pg-4F. The corresponding copolymer chains containing ethyl ester groups (species A′_*x*,*y*_) were also revealed in accord to the mass spectrum of the initial HA sample. The mass spectra revealed also the presence of unfunctionalized oligosaccharides, in particular in the case of the HA–FA–Pg-3F and the HA–FA–Pg-4F samples (Fig. 2). Thus, the MALDI mass spectra confirmed the formation of the reactive functionalized oligosaccharides.

### Structure of HA–FA–Pg and HA–FA–NEG–OA derivatives

The structure of HA–FA–Pg and HA–FA–NEG–OA derivatives was investigated by ^1^H and ^13^C NMR spectroscopic studies using D_2_O as the solvent. A comparative analysis was performed on the ^1^H NMR spectra obtained with HA–FA–Pg samples prepared by using different stoichiometric ratio between 1 and HA (*i.e.*1/HA ratio from 50 to 12.5%, see Table 1). In particular, two HA–FA–Pg samples were selected on the basis of their grafting degree spanning from 35% (HA–FA–Pg-2F) to 10% (HA–FA–Pg-2F) in comparison with the previously published sample HA–FA–Pg-3F showing an intermediate grafting degree (*i.e.* around 20%).^24^ The ^1^H NMR experiments (Fig. 3) confirmed the successful coupling between HA and 1 in our reaction conditions.

The ^1^H NMR spectra indeed showed, beside the typical profile of HA in the up-field region, signals attributed to the propargylated FA residues in the down-field region of the recorded spectra. Moreover, the relative intensity of the signals in the aromatic region increased from HA–FA–Pg-4F (grafting degree around 10%) to HA–FA–Pg-3F (grafting degree around 20%) and HA–FA–Pg-2F (grafting degree around 35%) with the increase of the grafting degree. This observation along with the broadness of the typical signal pattern in the aromatic region supported further the occurrence of the functionalization.

The more resolved spectral lines allowed a more detailed comparison of the ^13^C NMR spectra (Fig. 4) to be done.

The assignment of the ^13^C NMR spectra of HA and HA–FA–Pg samples was performed as previously described.^24^ In agreement with that observed in the analysis of ^1^H NMR spectra, the relative intensity of the signals attributed to propargylated FA residues increased with the increase of the grafting degree, which appeared to play a negligible role in modulating the shoulder of the signals attributed to anomeric C – a carbon atom of HA.

In order to evaluate the occurrence of the click chemistry coupling between the selected HA–FA–Pg samples (*i.e.*HA–FA–Pg-2F and HA–FA–Pg-3F) leading HA–FA–NEG–OA derivatives (*i.e.*HA–FA–NEG–OA–8 and HA–FA–NEG–OA–4, respectively), the ^1^H and ^13^C NMR spectra of HA–FA–NEG–OA–4 were analyzed in comparison with those of its synthetic precursor and HA–FA–Pg-3F (Fig. 5 and 6) and of the model compound FA–NEG–OA, which was extensively characterized by means 1D and 2D NMR techniques. In this way, we were able to assign all the signals of the complex spectra obtained with HA–FA–NEG–OA materials (see ESI†).

In fact, the ^1^H NMR spectrum of the HA–FA–NEG–OA–4 material (Fig. 5) appeared to be composed by a multitude of signals deriving from the integration of discrete signal patterns relevant to the different components of the material.

Interestingly, the disappearance of the peak at 2.91 ppm attributed to acetylene proton of HA–FA–Pg-3F and the appearance of a new peak at 8.02 in the spectrum of HA–FA–NEG–OA–4 material supported the transformation of the alkyne moiety into the triazole one as it usually occurs in CuAAC coupling reaction. Therefore, this observation was considered to be a strong evidence of the structure of FA–NEG–OA-4 material. Furthermore, a significant line broadening was observed in the signals attributed to the aromatic and acrylic protons of FA moiety.

Similarly, the ^13^C NMR spectrum of the HA–FA–NEG–OA–4 material (Fig. 6) resulted by the combination of the signal patterns relevant to the different components of the material. Among them, the signals attributed to the low molecular weight HA ribbons and to NEG side chains dominated the spectrum, with the one attributed oleic acid moiety playing a relatively minor role, and the ones belonging to the aromatic carbon atoms of ferulate component being lost in the baseline modulations. In agreement with previous observations,^24^ we assumed that owing to its role of linker between the two macromolecular components (*i.e.*HA and OEG–OA), the FA moiety suffered from a reduced mobility, which could produce a pronounced line broadening as already observed in the ^1^H NMR spectrum in Fig. 5.

Nevertheless, the persistence of the signal attributed to the ferulate methoxy C-A (at about 55.6 ppm) and the apparent shift of both the one attributed to the ferulate propargyl C-B (at about 56.4 ppm) and the one attributed to the ferulate propargyl carbons C-D and C-C (at about 76 ppm) were assumed to confirm the occurrence of the CuAAC coupling reaction.

**Fig. 7:**
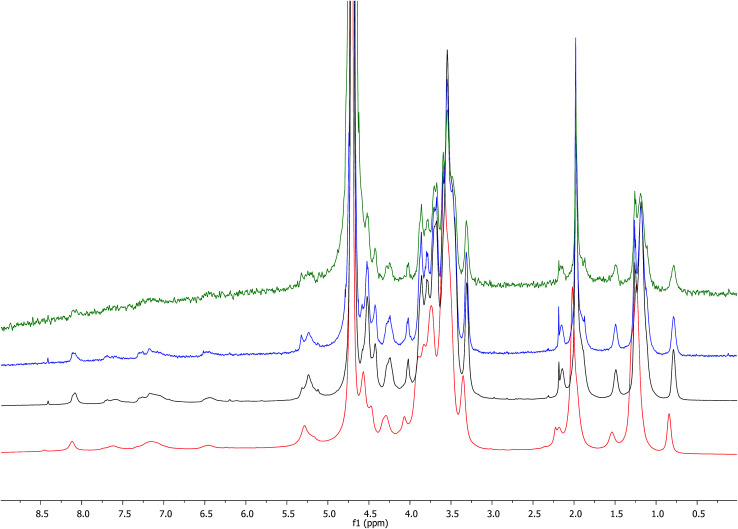
Comparison of ^1^H NMR spectra (600 MHz) obtained with increasing concentrations of HA–FA–NEG–OA–4 derivative in D_2_O (red: 25 mg mL^−1^, black: 2.5 mg mL^−1^, blue: 0.25 mg mL^−1^, green: 0.025 mg mL^−1^).

The comparison of the ^1^H NMR spectra obtained with increasing concentrations of HA–FA–NEG–OA–4 in D_2_O (Fig. 7) showed a gradual broadening, which suggested a tendency towards aggregation of this material.

**Fig. 8:**
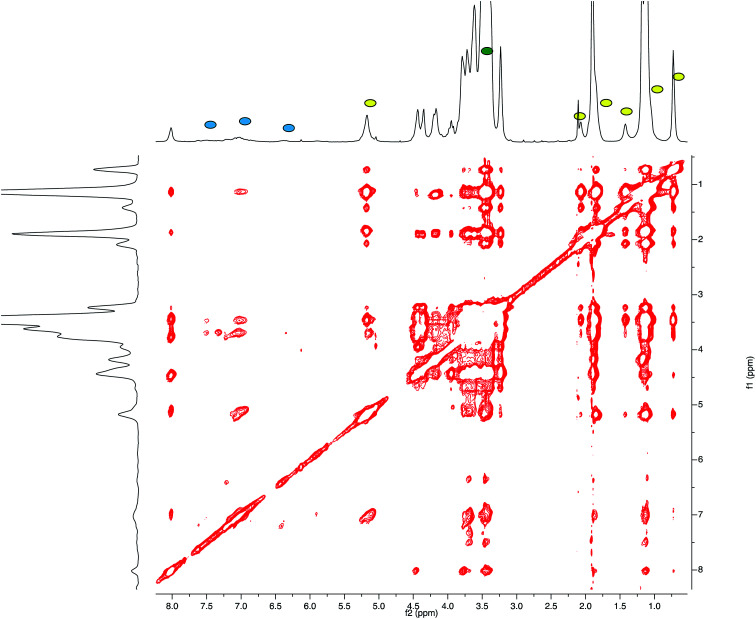
High field (900 MHz TCI cryo) NOESY spectrum (mixing time 200 ms) performed on the water (*i.e.* D_2_O, with water suppression) dispersions of HA–FA–NEG–OA–4 material.

This result led us to assume the formation of colloidal water dispersions with HA–FA–NEG–OA–4 in the form of micelles with a hydrophobic core constituted by the oleic residues. The high field (900 MHz) NOESY spectrum (Fig. 8) performed on the water dispersions of the material showed the presence of strong dipolar interactions among different portions of the macromolecules, which should be far in extended conformations, but were evidently near from each other in the 3D space.

This result suggested the existence of a strong entanglement of the side chain components (both the lipophilic oleic one and the amphiphilic NEG one) in the core of the micelles, which exposed on their surface the hydrophilic side of the hyaluronan ribbon as suggested by the apparent lack of dipolar interactions of the acetamide methyl group (sharp singlet at 1.92 ppm) with the side chain components.

The relative sign of cross-peaks in a NOESY spectrum depends on the rotational correlation time, *i.e.* if the rotational correlation time of the molecules is long, the diagonal and cross-peaks have same signs.^30^ This result allowed assertion that the HA–FA–NEG–OA–4 experienced a slower motion in solutions confirming the aggregation processes.

### Dynamic light scattering (DLS) characterization of HA–FA–NEG–OA derivatives in water

**Fig. 9:**
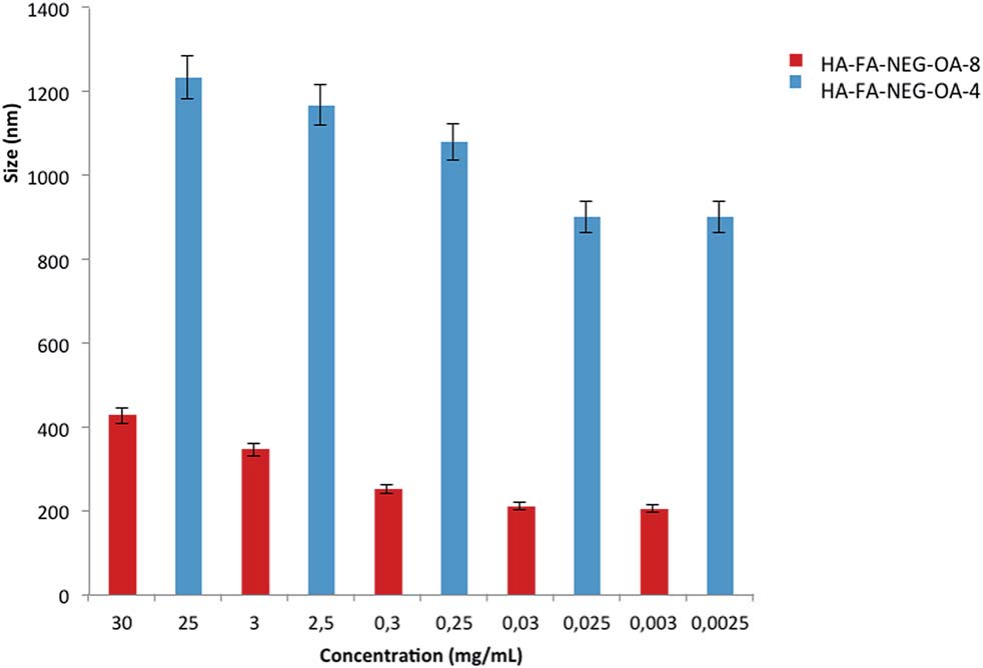
Mean particle size of HA–FA–NEG–OA material *versus* concentration of the water dispersions.

The aim of these experiments was to study the behavior of HA–FA–NEG–OA materials in water solution in relation to the variation of size with respect to concentration. The presence of oleic acid could lead to the formation of spherical or more probably worm-like micelles in solution. Fig. 9 reports the DLS data for HA–FA–NEG–OA materials.

At high concentration the HA–FA–NEG–OA–4 forms very large aggregates, whose polydispersity index (PDI) was reasonable even if quite high. In this case, the distribution curve obtained from Laplace reversal for such large diameter values may contain artifacts. The aggregate size analysis was, furthermore, limited to the restrictions of DLS, where the aggregate must remain small enough for Brownian forces to dominate gravitational force, preventing sedimentation, which for polymeric particles is generally considered to be 1000 nm.^31^ The high polydispersity indexes revealed that the systems were not perfectly monodispersed. The size decrease with concentration and this experimental data highlight that the macromolecule aggregate structure was similar to spherical or a worm-like micelles.

HA–FA–NEG–OA–8 at the maximum tested concentration of 30 mg mL^−1^ had two main distributions, whose data were not, however, very reproducible. This is typical of polymeric solutions in which macromolecules do not have a well-defined form. HA–FA–NEG–OA–8 produced smaller aggregate than HA–FA–NEG–OA–4. However, these experimental data suggest that the both HA–FA–NEG–OA materials in solution were able to form aggregates such as spherical or worm-like micelles.

### Optical properties of HA–FA–Pg and HA–FA–NEG–OA derivatives

The absorption/emission features of HA–FA–Pg and HA–FA–NEG–OA derivatives were characterized in comparison with those of model compounds 4–6^24^ and FA–NEG–OA containing the ferulate fluorophore (Table 3).

**Table 3:** Photophysical properties of 4–6, FA–NEG–OA, and polymeric materials HA–FA–Pg and HA–FA–NEG–OA

Compd	Solution^a^^,^^b^^,^^c^			Solid^d^^,^^e^	
	*λ*_ab_ (nm)	*λ*_em_ (nm)	PLQY (%)	*λ*_em_ (nm)	PLQY (%)
6	290, 320^a^^,^^b^	405^b^	0.34^b^	430^e^ (450^d^)	<0.1
4	290, 320^a^^,^^b^	390^a^ (410^b^)	0.24^a^ (0.34^b^)	370^e^ (390^d^)	4.4
FA–NEG–OA	290, 320^a^	400^a^	0.28^a^		
5	290, 320^a^	390^a^	0.24^a^	400^e^	<0.1
HA–FA–Pg-2F	290, 320^c^	450^c^	0.68^c^	420^e^	7.6
HA–FA–Pg-3F	290, 320^c^	445^c^	0.75^c^	420^e^	5.5
HA–FA–Pg-4F	290, 320^c^	445^c^	0.83^c^	420^e^	7.3
HA–FA–NEG–OA–4	290, 320^c^	440^c^	0.79^c^	420^e^	2.9
HA–FA–NEG–OA–8	290, 320^c^	430^c^	0.87^c^	415^e^	4.8

a Dichloromethane.

b Methanol.

c Water.

d Spin.

e Cast.

The absorption/emission features of ferulic acid have been previously demonstrated to be affected by solvent polarity and pH. In fact, FA possesses in its structure two acidic moiety showing well distinct p*K*_a_ values (4.4 and 9.0, see ref. 5). The neutral FA molecule is barely fluorescent, whereas the singly-ionized form is twofold more fluorescent (excitation maximum at 290–310 nm and emission maximum around 420 nm), and the doubly-ionized one is the most emissive specie (at pH = 10, the excitation maximum was shifted to 345 nm and emission maximum to 470 nm, see ref. 5). Furthermore, the decrease in solvent polarity was reported to produce decreases in the excitation, emission, and Stokes shift of FA solutions (*i.e.* the blue-green fluorescence was quenched by 80% in chloroform with respect to water, see ref. 5).

**Fig. 10:**
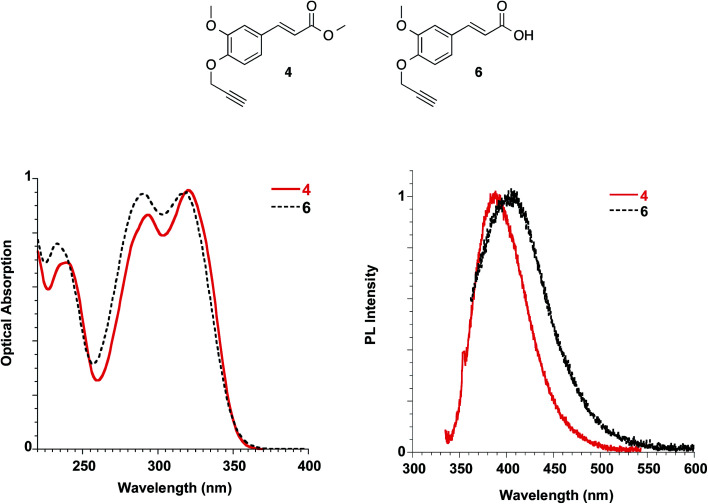
Absorption (left) and emission (right) spectra of ester 4 in dichloromethane and acid 6 in methanol at 10^−5^ M.

By the propargylation of the phenolic OH of FA as in compound 6, the less acidic group of FA is lost, and compound 6 shows absorption maxima similar to those reported for FA and slightly sensitive to solvent polarity (methanol: 235, 290 and 318 nm Fig. 10; dichloromethane: 238, 295 and 324 nm, see ESI†). On the other hand, the corresponding ester 4, lacking both the ionisable groups, shows very similar absorption spectra in the solvents with well-distinct polarities such as dichloromethane and methanol, with bands peaked at 238, 292 and 319 nm (see Fig. 10 and ESI†).

The molar extinction coefficient of ester 4 (*i.e.* 21 817 L mol^−1^ cm^−1^) was found to be well related to the one described for soyamide ferulate (*i.e.* 19 705 L mol^−1^ cm^−1^).^32^

**Fig. 11:**
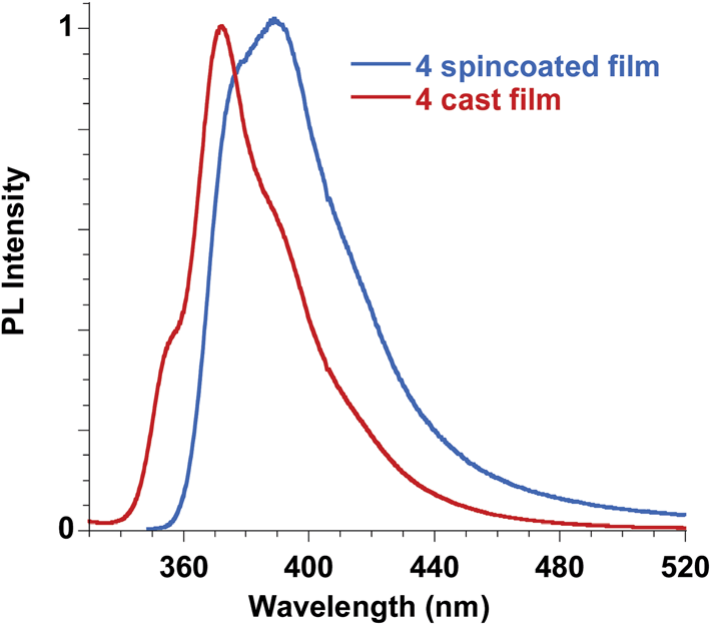
Emission spectra of ester 4 in the solid state, film obtained by spin coating compared to the film obtained by casting.

Ester 4 and the corresponding acid 6 showed similar emissive features in solution with PL maxima centred at 410 nm and 406 nm in methanol for 4 and 6, respectively, while the spectrum shifted at 388 nm for 4 in dichloromethane, in agreement with a reduction of the Stokes shift in solvents with lower polarities, as already reported for FA solutions. They were weakly emissive in solution (PLQY = 0.24% for ester 4 in dichloromethane and 0.34% for both ester 4 and acid 6 in methanol), but a significant increase in the PLQY (*i.e.* 4.4%) was observed in the films obtained with ester 4 while the emission of acid 6 in the solid state is very low (see Table 3, Fig. 11).

In order to better understand the emissive properties of the two compounds 4 and 6 we cooled their diluted solutions down to 77 K in order to block the molecular motions often responsible for the lack of emission of aggregation-induced emission (AIE) molecules in solution.^33,34^ We observed, for both compounds 4 and 6, a strong increase (a factor of about 50–80) in the emission intensity when the solutions are frozen at 77 K (see ESI†). This result demonstrates that, by restricting their molecular motions, both 4 and 6 compounds emit strongly, while upon aggregation only compound 4 displays a sensitive emission enhancement. This fact evidences the importance of a proper molecular packing for the exploitation of the AIE properties of this class of fluorogens, as observed for some closely related cinnamic derivatives.^35^ Therefore, compound 4 showed a typical AIE behavior,^36,37^ with photoluminescence quantum yields in the solid state increased by one order of magnitude with respect to the corresponding values measured in solution. Furthermore, we note that the emission spectra in the solid state depend on the film deposition procedure. When the film is obtained by casting the solution a better organization of the molecules is usually obtained with respect to the spincoating deposition, which causes a rapid evaporation of the solvent. As a result, the emission of the cast film displays a sharper and better structured spectrum while that of the spincoated film is closer to the solution one.

**Fig. 12:**
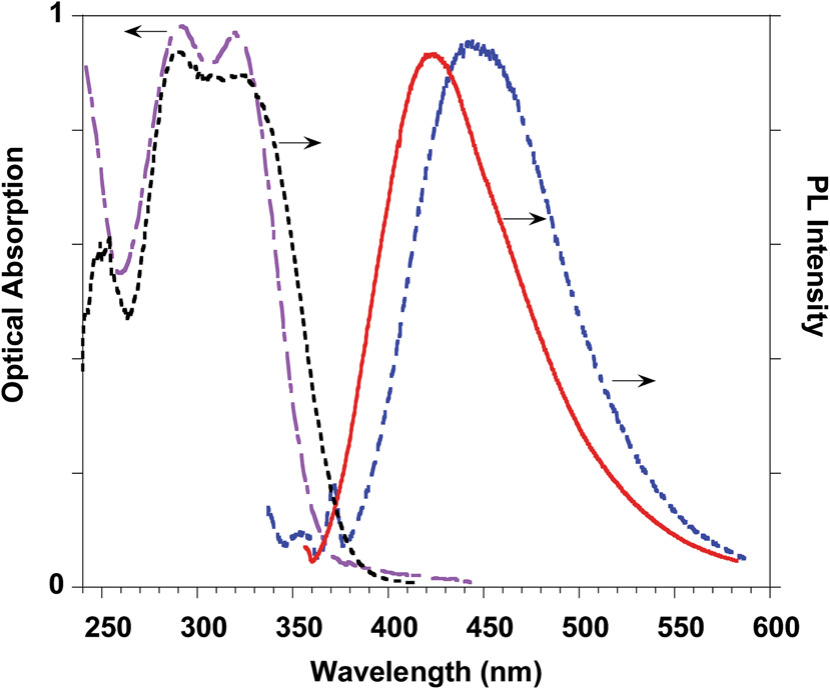
HA–FA–Pg-3F optical properties: absorption (pink dashed-dotted line) and emission (blue dashed line) spectra in water solution. PL (red line) and PL excitation (black dotted line) spectra of the solid state cast film.

This result stimulated our interest in evaluating the effects of the introduction of ferulate fluorogen in the rigid HA backbone as in HA–FA–Pg graft copolymers. The photophysical features of three HA–FA–Pg samples showing different grafting degrees were investigated both in the water solutions and in the solid state. The absorption spectra of the polymeric materials were dominated by the absorption of the ferulate fluorophores with maxima perfectly matching with those of ester 4 (290 and 320 nm), whereas a significant red shift was observed in the emission spectra recorded with the water solutions of HA–FA–Pg, as expected due to the high polarity of the solvent (Table 3, Fig. 12).

The PLQY values measured with the water solutions of HA–FA–Pg samples resulted higher (in the range of 0.68–0.83%) than of the corresponding value obtained with the solution of ester 4 in dichloromethane (0.24%). This effect was retained also when HA–FA–NEG–OA were compared with the corresponding model compounds 5 and FA–NEG–OA and could be rationalized with the decrease of the mobility of the ferulate fluorogen as suggested by NMR studies.

A further significant increase in the PLQY was observed in the solid-state samples (*i.e.* cast films) of the polymeric materials, which showed PLQY values (in the range 2.9–7.6%) sufficiently promising for a further characterization of these materials in view of their potential biomedical applications.

### Interactions of HA–FA–NEG–OA derivatives with phospholipid bilayers

Owing to their peculiar architecture, HA–FA–NEG–OA derivatives were assumed to interact with cell membranes. In order to evaluate this hypothesis, preliminary studies were performed by using unilamellar liposomes as simplified models of cell membranes. Liposomes were synthesized with 1,2-dioleoyl-*sn*-glycerophosphocholine (DOPC) and 1,2-dioleoyl-*sn*-glycerophosphoethanolamine (DOPE), which are commonly used in zwitterionic liposomes,^38,39^ since they form a fluid bilayer at room temperature. Furthermore, the phosphocholine and phosphoethanolamine are the most abundant polar head types in the outer cell membrane.^40^

Unilamellar vesicles showing sub-micrometer dimensions (SUV) were prepared by standard procedures, and the mean diameters, polydispersity indexes and *ζ*-potential of the DOPC/DOPE liposomes with and without HA–FA–NEG–OA–8 (Table 4) indicated that insertion of the material into liposomes led to an increase in the mean diameter of vesicles. The vesicle diameter changed significantly after the addition (143.9 ± 6.8 nm). The low polydispersity indexes of plain and loaded systems revealed that liposomes were not substantially altered by interaction with HA–FA–NEG–OA–8. Indeed, synthesized liposomes remained fairly monodispersed.

**Table 4:** Mean particle size, polydispersity index, and surface charge of pure and HA–FA–NEG–OA–8 loaded liposomes obtained by extrusion through 100 nm polycarbonate membranes

Liposome composition	Size ± SD (nm)	PDI	*ζ* potential ± SD (mV)
DOPC/DOPE	105.7 ± 11.0	0.16	−13.6 ± 4.8
DOPC/DOPE + HA–FA–NEG–OA–8	143.9 ± 6.8	0.18	−41.0 ± 10.3

Plain DOPC/DOPE liposomes had a small negative *ζ*-potential, though the net polar head charge of zwitterionic phospholipids is zero. The insertion of HA–FA–NEG–OA–8 changes the zeta potential to more negative values (−41.0 mV) suggesting that the system is chemically stable and no aggregation or flocculation processes may occur in the solution.

Thus, the photophysical characterization of HA–FA–NEG–OA–8 derivative was deepened in the phospholipid environment, both in the SUV dispersions in water and in the solid state of the cast films. The results (see ESI†) suggested that the interaction of HA–FA–NEG–OA–8 derivative with phospholipid bilayers produced negligible effects in the photophysical features of HA–FA–NEG–OA derivatives maintaining the AIE properties of the fluorogen.

### *In vitro* cytotoxicity and cytocompatibility: cell viability

**Fig. 13:**
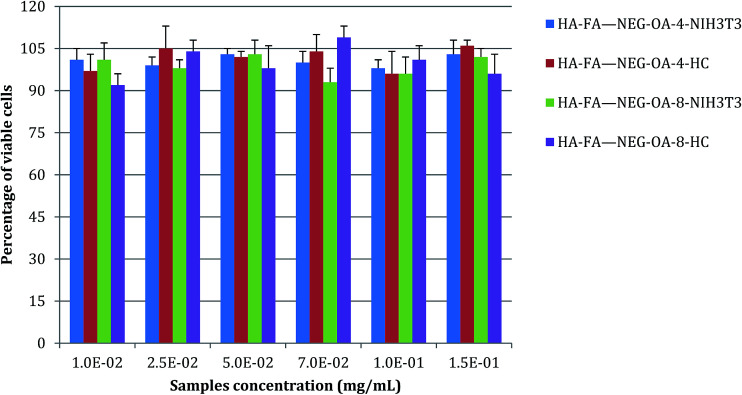
NIH3T3 and HC viability after 24 h of contact with different concentrations of HA–FA–NEG–OA–4 and HA–FA–NEG–OA–8 materials as determined by the neutral red uptake. Data are mean ± SD of six replicates. Values are not statistically different *versus* negative control (complete medium).

Non-confluent adhered fibroblasts and chondrocytes were incubated with different concentrations of HA–FA–NEG–OA materials. Cells were analysed after 24 hours and the results are reported in Fig. 13. The presence of both HA–FA–NEG–OA materials, at any of the tested concentrations, did not affect NIH3T3 and HC viability in comparison to the control (culture medium) and the samples met requirements of ISO 10993-5 standard for cytocompatibility of materials, *i.e.*, cells cultured in the presence of HA–FA–NEG–OA derivatives possessed over 70% of viability of cells cultured in the standard medium.

Moreover, the results obtained demonstrated the high degree of *in vitro* cytocompatibility of the test materials which did not interfere with cell viability of both immortalized cells, such as 3T3 fibroblasts, and primary human cells, such as chondrocytes.

### Fluorescence imaging

**Fig. 14:**
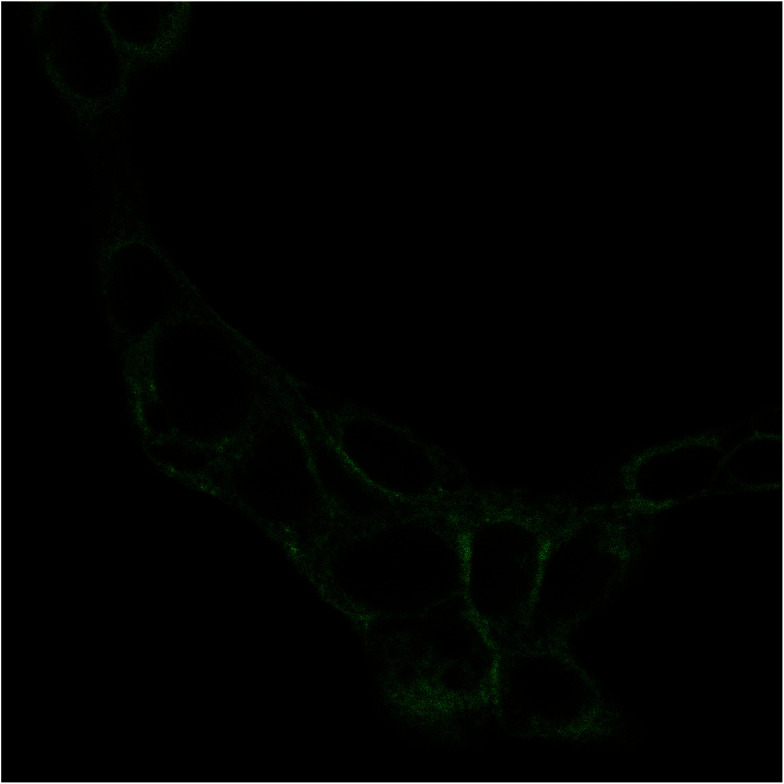
Fluorescence image of NIH3T3 fibroblast after 15 min of contact with HA–FA–NEG–OA–4 material. Cells were cultured on glass coverslips and imaged on the same slides previous treatment with 4% paraformaldehyde.

The internalization of HA–FA–NEG–OA–4 derivative by NIH3T3 cells was evaluated in preliminary studies by fluorescent microscopy. Analysis of fluorescence signal from the NIH3T3 fibroblasts cultured in medium containing HA–FA–NEG–OA–4 material revealed a marked fluorescence distributed in the cells cytoplasm (Fig. 14). These results together with analysis of cell viability data, shows that in the case of both immortalized and primary cells, the presence of HA–FA–NEG–OA–4 do not affect cell growth.

**Fig. 15:**
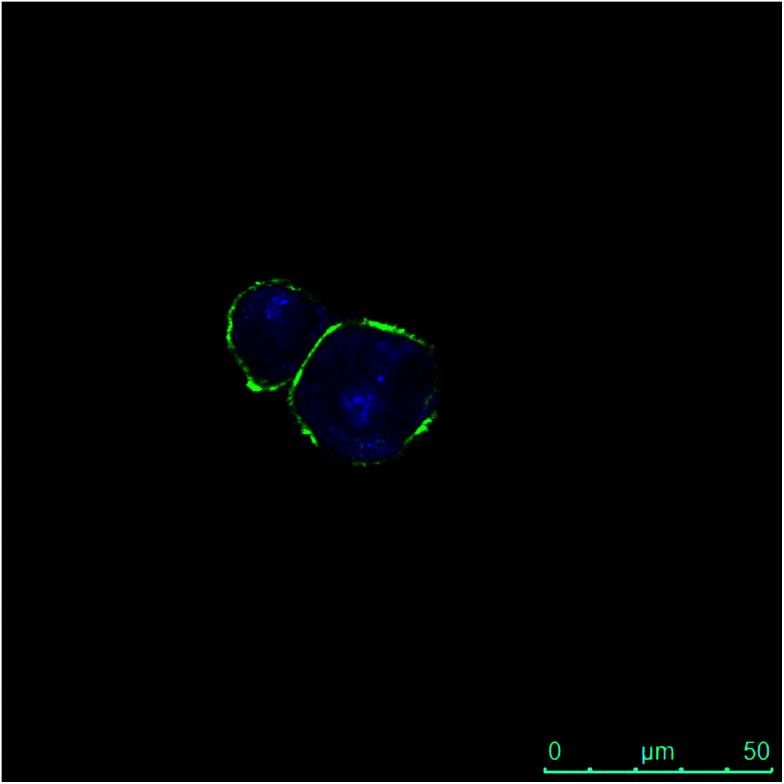
Fluorescence image of PANC-1 cells after 30 min of contact with HA–FA–NEG–OA–8 material and after 2 h internalization. The green fluorescence is due to the FITC-conjugated lectin interacting with membranes, whereas the blue emission is produced by HA–FA–NEG–OA–8 material internalized into the cytoplasm.

Moreover, the internalization of both the polymeric materials (*i.e.*HA–FA–NEG–OA–4 and HA–FA–NEG–OA–8) was further confirmed by confocal microscopy in PANC-1 cells with membrane labelling by means of FITC-conjugated lectin in order to better visualize the cellular surface (Fig. 15).

In fact, a clear fluorescence signal was observed in the cytoplasm of the cells treated with HA–FA–NEG–OA–4 or HA–FA–NEG–OA–8 materials that was attributed to ferulic fluorogen internalized into PANC-1 cells after 30 min of contact and 2 h of internalization. This result suggests for HA–FA–NEG–OA materials potential applications in intracellular delivery.

## Conclusions

In conclusion, we have developed a technology platform based on two natural compounds from biorenewable resources, namely low molecular weight HA playing the role of the macromolecular carrier, and FA playing the role of the aggregation-induced emission (AIE) fluorophore bearing clickable propargyl groups. Thus, a short series of hyaluronan-based graft copolymers (HA–FA–Pg) were designed and synthesized to show different grafting degree values and their optical properties were characterized in comparison with reference compounds containing the same ferulate fluorophore. Interestingly, these studies showed that the push–pull structure of the cinnamic scaffold of ferulate fluorophore was quite sensitive to the restriction of intramolecular motions (RIM) phenomenon, thus showing fluorogenic properties. In fact, model compound 4 showed a typical aggregation-induced emission behavior, and the introduction of ferulate fluorogen in the rigid HA backbone as in HA–FA–Pg graft copolymers led to PLQY values in the solutions higher than of the corresponding value obtained with the solution of model compound 4 in dichloromethane.

In the attempt to demonstrate the usefulness of the technology platform, the propargyl groups of HA–FA–Pg derivatives were exploited in the coupling with a third natural component from biorenewable resources (*i.e.* oleic acid) through a biocompatible spacer composed of nine monomeric units of ethylene glycol. The resulting HA–FA–NEG–OA derivatives showed self-assembling capabilities in aqueous environment and for this reason were characterized as potential biocompatible self-assembled aggregate forming material for biomedical applications. In particular, HA–FA–NEG–OA derivatives were demonstrated to interact with phospholipid bilayers both in liposomes and living cells retaining the fluorogenic properties and showing a high degree of cytocompatibility.

On the basis of its high modularity, we envisioned for this technology platform a broad applicability. In fact, a broad range of polymeric carriers can be used beside to different fatty acid residues and oligo(ethylene glycol) spacers, with ferulate playing the central role of natural small molecule fluorogenic clickable linker.

## Conflicts of interest

The authors declare no competing financial interest.

## Supplementary Material

RA-008-C7RA12543G-s001
