# Supplementary material for: Hyaluronan-based graft copolymers bearing aggregation-induced emission fluorogens
Source: RSC Adv. 2018 Feb 6;8(11):5864–81. doi: 10.1039/c7ra12543g (PMC9078255; doi:10.1039/c7ra12543g)
Supplement: RA-008-C7RA12543G-s001 [file RA-008-C7RA12543G-s001.pdf]

Electronic Supplementary Material (ESI) for RSC Advances.
This journal is © The Royal Society of Chemistry 2018

Electronic Supplementary Information

Hyaluronan-Based Graft Copolymers Bearing Aggregation-Induced Emission Fluorogens

Andrea Cappelli,[*,a] Marco Paolino,[a] Annalisa Reale,[a] Vincenzo Razzano,[a] Giorgio Grisci,[a] Germano Giuliani,[a] Alessandro Donati,[a] Claudia Bonechi,[a] Stefania Lamponi,[a] Raniero Mendichi,[b] Salvatore Battiato,[c] Filippo Samperi,[c] Francesco Makovec,[d] Mariano Licciardi,[e,f] Lorenzo Depau,[g] Chiara Botta.[b]

Content:

Synthetic procedures for the preparation of azide **3** and of model compounds **5** and **FA-NEG-OA**;

^{1}H NMR spectrum of **HA-FA-NEG-OA**-8 compared with that of **HA-FA-NEG-OA**-4;

^{13}C NMR spectrum obtained with **HA-FA-NEG-OA**-4 derivative compared with those obtained with **HA**, **HA-FA-Pg**-3F, and model compound **FA-NEG-OA**;

Comparison of differential MWD of **HA** and four **HA-FA-Pg** derivatives;

Evaluation of NIH3T3 and HC viability;

Absorption spectra of compounds **4** and **6**;

Comparison of the photophysical properties of **HA-FA-NEG-OA**-8 in different environments;

Fluorescence image of PANC-1 cells after 30 min of contact with **HA-FA-NEG-OA**-4 material and after 2 h internalization.

Chemistry

The preparation of azide intermediate **3** and of model compounds **5** and **FA-NEG-OA** was carried out as shown in Scheme ESI-1.

Scheme ESI-1. Synthesis of azide **3** and of model compounds **5** and **FA-NEG-OA**.

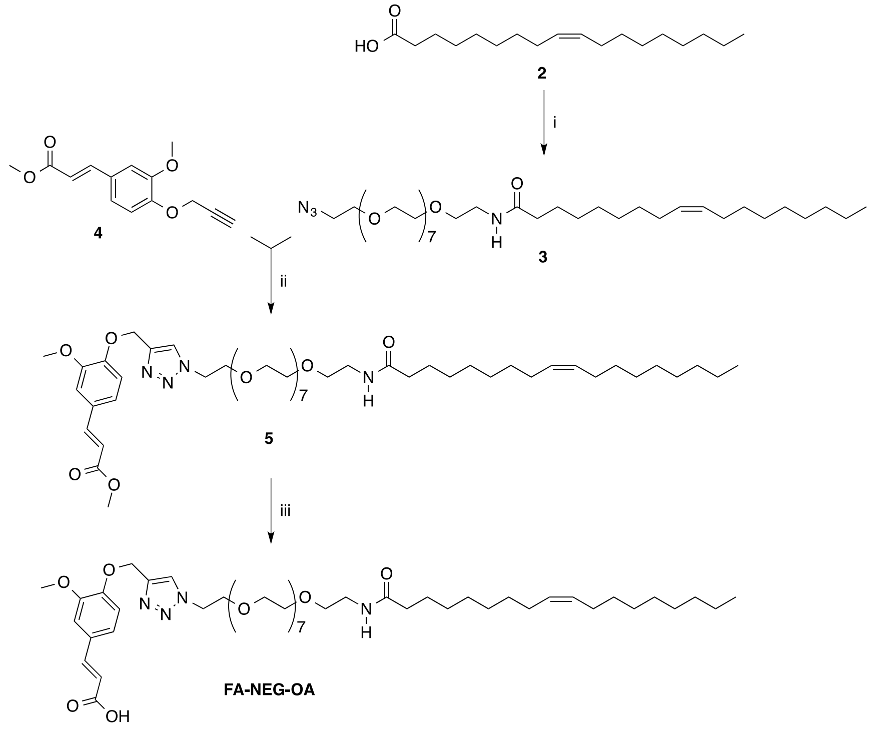

Reagents: (i) CDI, THF, $N_3CH_2CH_2(OCH_2CH_2)_8NH_2$; (ii) $CuSO_4$, sodium ascorbate, *tert*-BuOH, H_2O; (iii) NaOH, H_2O, C_2H_5OH.

Oleic acid (**2**) was activated by reaction with one equivalent of 1,1'-carbonyldiimidazole (CDI) in THF and then reacted with commercially available O-(2-aminoethyl)-O′-(2-azidoethyl)heptaethylene glycol to afford alpha-azido-omega-oleic amide nona(ethylene glycol) (azido-**NEG-OA**) **3**, which was also used in the preparation of model compounds **5** and **FA-NEG-OA**. In particular, the CuAAC reaction of propargyloxy derivative **4**[1] with (azido-**NEG-OA**) **3** gave model compound **5,** which was promptly hydrolyzed in basic conditions to afford acid **FA-NEG-OA.**

Synthetic procedures

Methods. All reagents and solvents were purchased from Sigma-Aldrich and were used as received. Merck silica gel 60 (230-400 mesh) was used for column chromatography. Merck TLC aluminum sheets, silica gel 60 F_{254} were used for TLC. NMR spectra were recorded with a Bruker DRX-400 AVANCE or a Bruker DRX-500 AVANCE spectrometer in the indicated solvents (TMS as internal standard): the values of the chemical shifts are expressed in ppm and the coupling constants (*J*) in Hz. An Agilent 1100 LC/MSD operating with an electrospray source was used in mass spectrometry experiments.

N-(26-Azido-3,6,9,12,15,18,21,24-octaoxahexacosyl)oleamide (3).

A mixture of oleic acid (**2**, 200 mg, 0.708 mmol) in dry THF (5.0 mL) containing CDI (115 mg, 0.709 mmol) was refluxed for 3 h and then a solution O-(2-aminoethyl)-O′-(2-azidoethyl)heptaethylene glycol (213 mg, 0.486 mmol) in dry THF was added. The resulting mixture was stirred overnight at room temperature and then concentrated under reduced pressure. Purification of the residue by flash chromatography with ethyl acetate-methanol (97:3) gave **3** as a pale yellow oil (305 mg, yield 89%). ^{1}H NMR (400 MHz, $CDCl_3$): 0.87 (t, J = 6.5, 3H), 1.22-1.36 (m, 20H), 1.57-1.65 (m, 2H), 1.96-2.04 (m, 4H), 2.16 (t, J = 7.5, 2H), 3.36-3.40 (m, 2H), 3.44 (q, J = 4.8, 2H), 3.54 (t, J = 4.9, 2H), 3.59-3.70 (m, 30H), 5.27-5.39 (m, 2H), 6.09 (br s, 1H). MS (ESI): *m*/*z* 725 (M + Na^+).

(*E*)-Methyl 3-[3-methoxy-4-[[1-[(*Z*)-28-oxo-3,6,9,12,15,18,21,24-octaoxa-27-azapentatetracont-36-enyl]-1*H*-1,2,3-triazol-4-yl]methoxy]phenyl]acrylate (5).

A round bottom 10 mL flask was charged under an inert atmosphere with *tert*-butanol (2.0 mL), water (2.0 mL), and a solution of $CuSO_4$ pentahydrate (12.5 mg, 0.050 mmol) in 0.50 mL of water. Then, a 1M solution of sodium ascorbate in water (0.50 mL) was added and 1.0 mL of the resulting mixture was used as the catalyst. A mixture of **3** (100 mg, 0.142 mmol) and **4**[1] (53 mg, 0.215

mmol) in 4.0 mL of *tert*-butanol and water (2.0 mL) was treated with the catalyst solution (2.7 mL) and the reaction mixture was stirred overnight at room temperature and then concentrated under reduced pressure. Purification of the residue by flash chromatography with ethyl acetate-methanol (9:1) as the eluent afforded **5** as a pale yellow oil (110 mg, yield 82%). ^{1}H NMR (400 MHz, $CDCl_3$): 0.86 (t, *J* = 6.9, 3H), 1.21-1.34 (m, 20H), 1.56-1.64 (m, 2H), 1.95-2.02 (m, 4H), 2.14 (t, *J* = 7.6, 2H), 3.42 (q, *J* = 4.9, 2H), 3.50-3.70 (m, 30H), 3.77 (s, 3H), 3.84-3.91 (m, 5H), 4.52 (t, *J* = 4.7, 2H), 5.28-5.36 (m, 4H), 6.08 (br s, 1H), 6.29 (d, *J* = 15.9, 1H), 7.00-7.13 (m, 3H), 7.60 (d, *J* = 15.9, 1H), 7.85 (br s, 1H). ^{13}C NMR (125 MHz, $CDCl_3$): 14.1, 22.7, 25.8, 27.2, 29.2, 29.3, 29.5, 29.8, 31.9, 36.7, 39.1, 50.7, 51.7, 55.9, 62.8, 69.3, 70.0, 70.2, 70.5, 110.1, 113.5, 115.8, 122.4, 124.7, 128.0, 129.8, 130.0, 143.6, 144.7, 149.6, 149.7, 167.6, 173.3. MS (ESI): *m*/*z* 972 (M + Na^+).

(*E*)-3-[3-Methoxy-4-[[1-[(*Z*)-28-oxo-3,6,9,12,15,18,21,24-octaoxa-27-azapentatetracont-36-enyl]-1*H*-1,2,3-triazol-4-yl]methoxy]phenyl]acrylic acid (FA-NEG-OA).

A mixture of ester **5** (100 mg, 0.105 mmol) in ethanol (3.0 mL) containing a 2N water solution of NaOH (0.42 mL) was stirred at room temperature for 3 days. The reaction mixture was then cooled at 0 °C, acidified with 3N HCl, and concentrated under reduced pressure. Purification of the residue by flash chromatography with chloroform-methanol (95:5) gave acid **FA-NEG-OA** as a pale yellow oil (78 mg, yield 79%). ^{1}H NMR (400 MHz, $CDCl_3$): 0.86 (t, *J* = 7.0, 3H), 1.21-1.36 (m, 20H), 1.56-1.64 (m, 2H), 1.94-2.03 (m, 4H), 2.16 (t, *J* = 7.5, 2H), 3.43 (q, *J* = 5.1, 2H), 3.51-3.63 (m, 30H), 3.84 (t, *J* = 5.0, 2H), 3.88 (s, 3H), 4.51 (t, *J* = 5.0, 2H), 5.29-5.36 (m, 4H), 6.27-6.32 (m, 2H), 7.04-7.08 (m, 3H), 7.65 (d, *J* = 15.9, 1H), 7.82 (s, 1H). ^{13}C NMR (125 MHz, $CDCl_3$): 14.1, 22.7, 25.8, 27.2, 29.2, 29.3, 29.5, 29.8, 31.9, 36.7, 39.2, 50.4, 55.9, 62.9, 69.4, 70.0, 70.2, 70.5, 110.2, 113.6, 115.5, 122.7, 124.4, 127.8, 129.8, 130.0, 143.5, 146.0, 149.7, 149.78, 169.8, 173.6. MS (ESI): *m*/*z* 958 (M + Na^+).

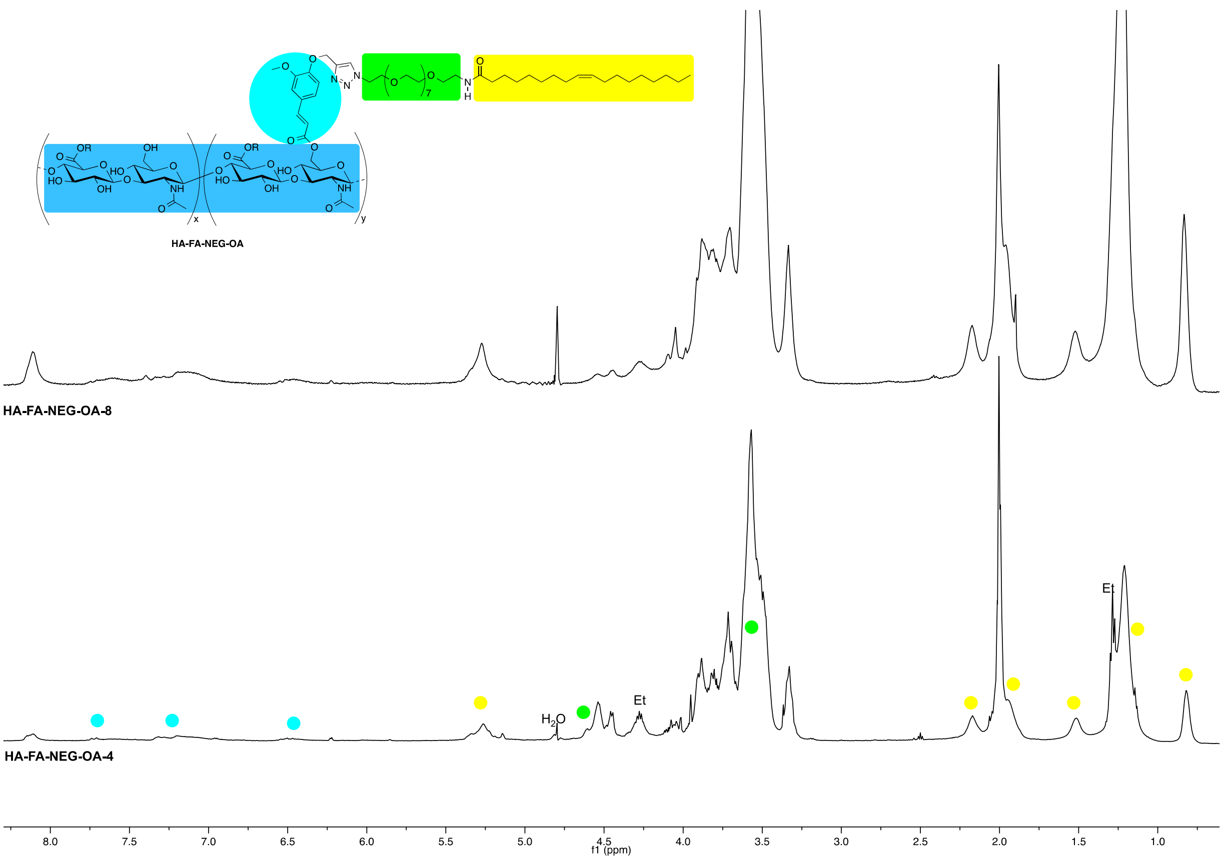

Figure ESI-1. Comparison of ^{1}H NMR spectra (500 MHz, D_2O, with water suppression) obtained with **HA-FA-NEG-OA**-8 material with that obtained with **HA-FA-NEG-OA**-4 one. In the spectrum of **HA-FA-NEG-OA**-4, Et labels indicate the signals of ethyl groups of the monomeric units showing R = C_2H_5.

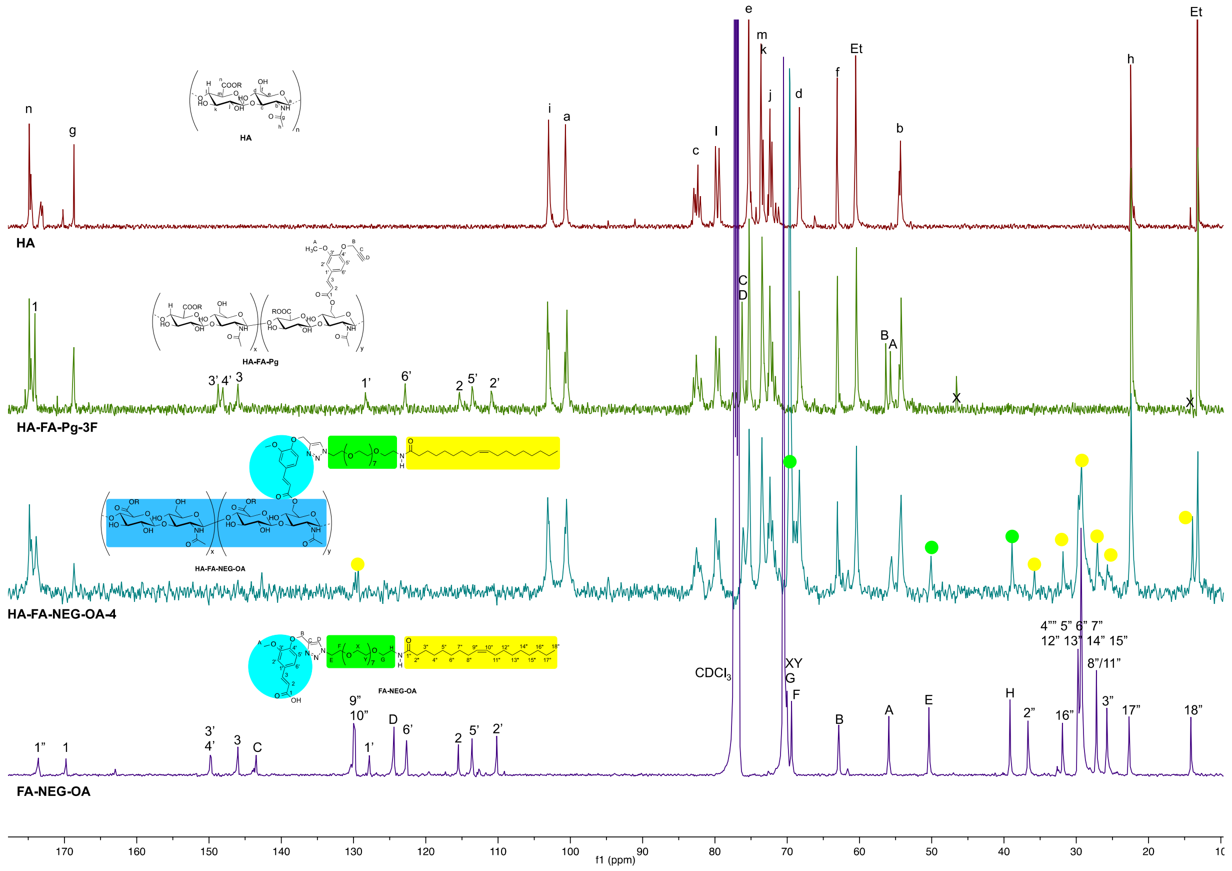

Figure ESI-2. Comparison of ^{13}C NMR spectra obtained with **HA-FA-NEG-OA**-4 derivative (125 MHz, D_2O) with those obtained with **HA**, **HA-FA-Pg**-3F, and model compound **FA-NEG-OA**. In the spectrum of **HA**, Et labels indicate the signals of ethyl groups of the monomeric units showing R = C_2H_5.

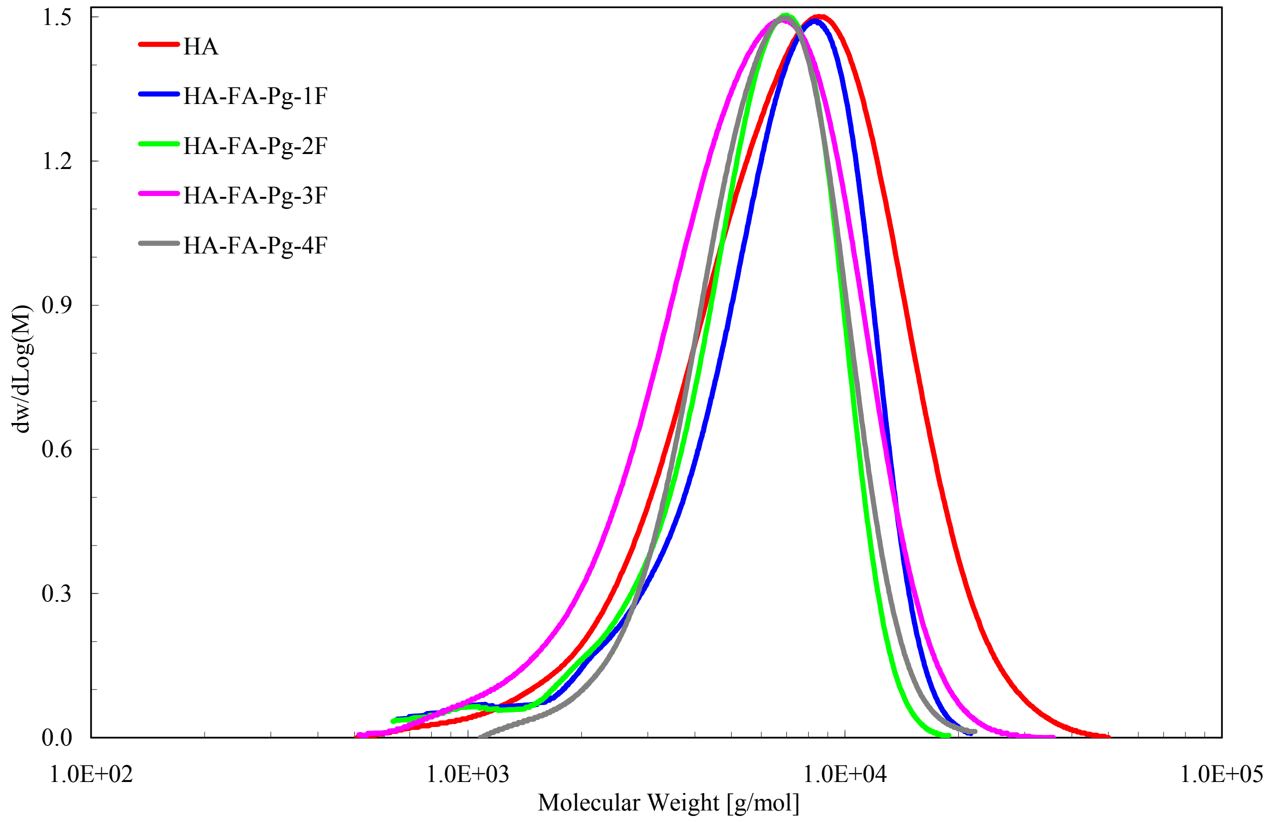

Figure ESI-3. Comparison of differential MWD of **HA** and four **HA-FA-Pg** derivatives.

Evaluation of NIH3T3 and HC viability

Cells (1.5 x 10^4) suspended in 1 mL of complete medium were seeded in each well of a 24 well round multidish and incubated at 37 °C in an atmosphere of 5% CO_2. Once reached the 50% of confluence (i.e. after 24 hours of culture), the culture medium was discharged and the following concentrations of **HA-FA-NEG-OA** materials were tested: 1.0x10^{-2}, 2.5x10^{-2}, 5x10^{-2}, 7x10^{-2}, 1x10^{-1}, 1.5x10^{-1} mg/mL.

All samples were set up in six replicates. Complete medium was used as negative control. After 24 hours of incubation, cell viability was evaluated by Neutral Red uptake, as follows. First, the following solutions were prepared in order to determine the percentage of viable cells:

1. Neutral Red (NR) stock solution: 0.33 g NR dye powder in 100 mL of sterile water;
2. NR medium: 1.0 mL NR stock solution+99.0 routine culture medium pre-warmed to 37 °C;
3. NR desorb solution: 1% glacial acetic acid solution + 50% ethanol + 49% water.

At the end of incubation, the routine culture medium was removed from each plate and the cells were carefully rinsed with 1 mL pre-warmed D-PBS 0.1M. Plates were then gently blotted with paper towels. 1.0 mL NR medium was added to each dish and further incubated at 37 °C, 95% humidity, 5.0% CO_2 for 3 h. The cells were checked during incubation for NR crystal formation. After incubation, the NR medium was removed and the cells were carefully rinsed with 1 mL pre-warmed D-PBS 0.1M. PBS was decanted and blotted from the dishes and exactly 1 mL NR desorb solution was added to each sample. Plates were placed on a shaker for 20-45 min to extract NR from the cells and form a homogeneous solution. During this step the samples were covered to protect them from light. Five min after removal from the shaker, absorbance was read at 540 nm with a UV/visible spectrophotometer (Lambda 25, Perkin Elmer).

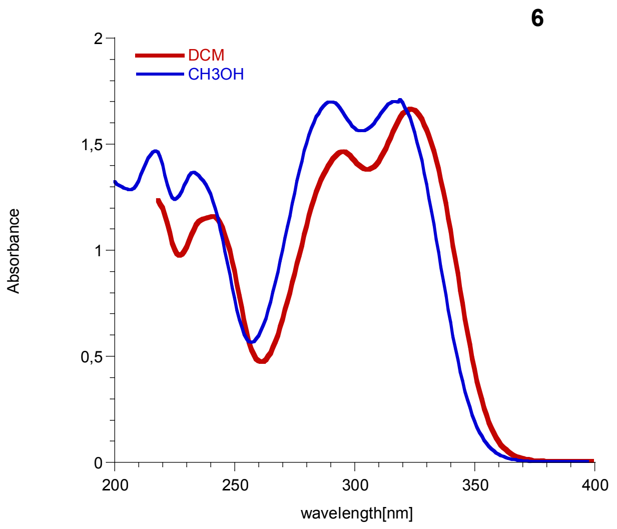

Figure ESI-4. Absorption spectra of **6** in dichloromethane and in methanol.

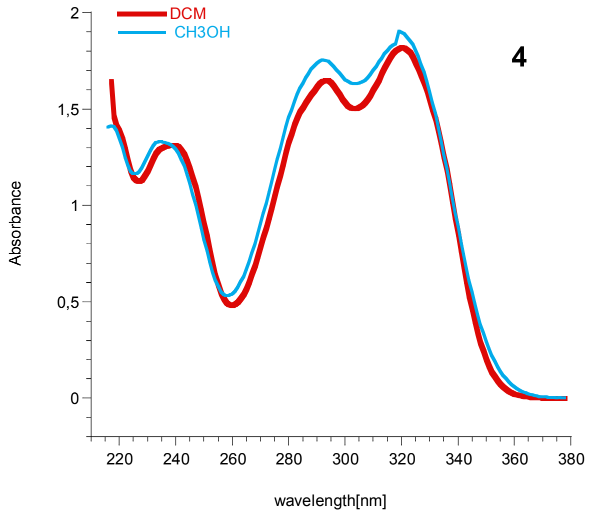

Figure ESI-5. Absorption spectra of **4** in dichloromethane and in methanol

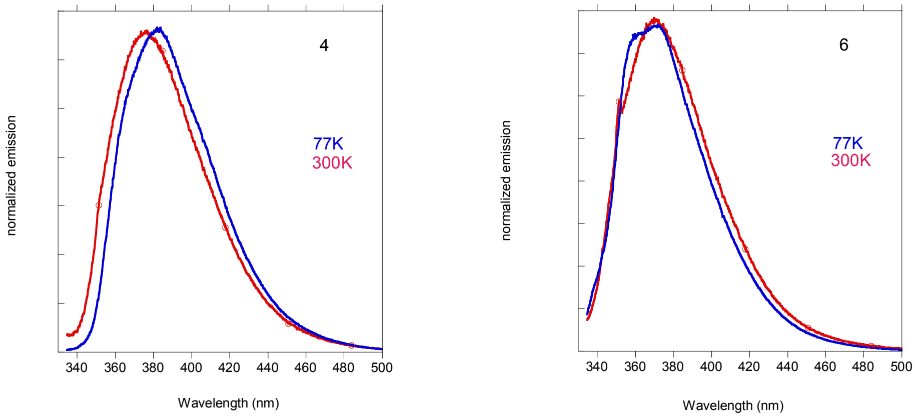

Figure ESI-6. Emission spectra of **4** and **6** in THF at room temperature and at 77K.
Photophysical properties of THF diluted (10^{-5}M) solutions:
4 QY 0.38%, abs 320 nm/em 375 nm (383 nm a 77K);
6 QY 0.39%, abs 318nm/em 370nm.

Table ESI-1. Comparison of the photophysical properties of **HA-FA-NEG-OA**-8 in different environments.

	water solution			solid	
Environment	λ_{ab} (nm)	λ_{em} (nm)	PLQY (%)	λ_{em} (nm)	PLQY (%)
Alone	290, 320	430	0.87	420	4.8
SUV	290, 320	430	0.82	410	5.0

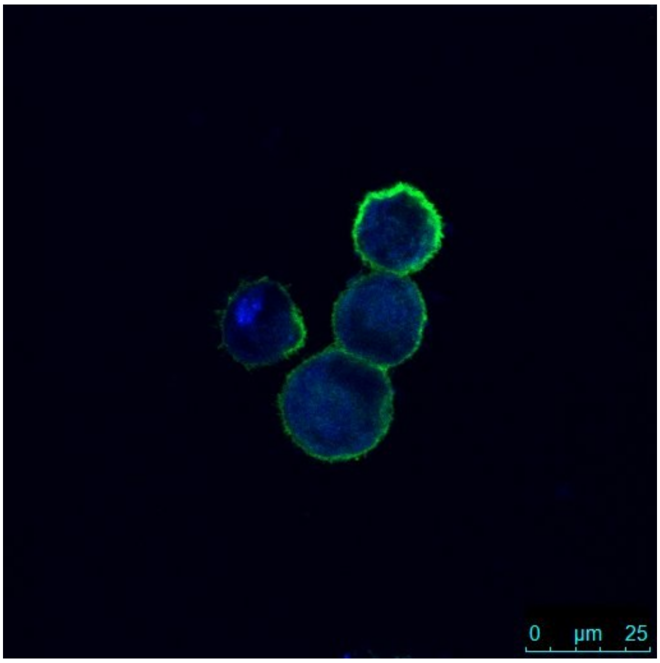

Figure ESI-7. Fluorescence image of PANC-1 cells after 30 min of contact with **HA-FA-NEG-OA**-4 material and after 2 h internalization. The green fluorescence is due to the FITC-conjugated lectin interacting with membranes, whereas the blue emission is produced by **HA-FA-NEG-OA**-4 material internalized into the cytoplasm.

References

1. A. Cappelli, M. Paolino, G. Grisci, V. Razzano, G. Giuliani, A. Donati, C. Bonechi, R. Mendichi, S. Battiato, F. Samperi, C. Scialabba, G. Giammona, F. Makovec, M. Licciardi, *Polym. Chem*. 2016, **7**, 6529-6544.
